# Stochasticity of Intranuclear Biochemical Reaction Processes Controls the Final Decision of Cell Fate Associated with DNA Damage

**DOI:** 10.1371/journal.pone.0101333

**Published:** 2014-07-08

**Authors:** Kazunari Iwamoto, Hiroyuki Hamada, Yukihiro Eguchi, Masahiro Okamoto

**Affiliations:** 1 Graduate school of Systems Life Sciences, Kyushu University, Fukuoka, Japan; 2 Japan Society for the Promotion of Science, Tokyo, Japan; 3 Department of Systems Life Sciences, Kyushu University, Fukuoka, Japan; 4 Department of Bioscience and Biotechnology, Faculty of Agriculture, Kyushu University, Fukuoka, Japan; 5 Synthetic Systems Biology Research Center, Kyushu University, Fukuoka, Japan; 6 Kyushu University Bio-Architecture Center, Kyushu University, Fukuoka, Japan; German Cancer Research Center, Germany

## Abstract

A massive integrative mathematical model of DNA double-strand break (DSB) generation, DSB repair system, p53 signaling network, and apoptosis induction pathway was constructed to explore the dominant factors of unknown criteria of cell fate decision. In the proposed model, intranuclear reactions were modeled as stochastic processes and cytoplasmic reactions as deterministic processes, and both reaction sets were simulated simultaneously. The simulated results at the single-cell level showed that the model generated several sustained oscillations (pulses) of p53, Mdm2, ATM, and Wip1, and cell-to-cell variability in the number of p53 pulses depended on IR intensity. In cell populations, the model generated damped p53 oscillations, and IR intensity affected the amplitude of the first p53 oscillation. Cells were then subjected to the same IR dose exhibiting apoptosis induction variability. These simulated results are in quantitative agreement with major biological findings observed in human breast cancer epithelial MCF7, NIH3T3, and fibrosarcoma cells, demonstrating that the proposed model was concededly biologically appropriate. Statistical analysis of the simulated results shows that the generation of multiple p53 pulses is a prerequisite for apoptosis induction. Furthermore, cells exhibited considerable individual variability in p53 dynamics, which correlated with intrinsic apoptosis induction. The simulated results based on the proposed model demonstrated that the stochasticity of intranuclear biochemical reaction processes controls the final decision of cell fate associated with DNA damage. Applying stochastic simulation to an exploration of intranuclear biochemical reaction processes is indispensable in enhancing the understanding of the dynamic characteristics of biological multi-layered systems of higher organisms.

## Introduction

The tumor suppressor gene p53 is activated in response to various stresses, including ionizing radiation (IR), and acts as a transcription factor to regulate expression of many other genes. The genes regulated by p53 induce multifarious cellular responses, e.g., cell cycle arrest, DNA repair, and programmed cell death (apoptosis) [1], [2]. These responses, which correspond to a sequence of biological events leading from p53 gene expression to apoptosis induction, are known as cell fate decision, and contribute to both growth inhibition of tumor cells and genetic homeostasis [3]. However, the cell fate decision mechanism applies unknown criteria to various stress intensities [4]. Because the fluctuation of criteria affects the efficiency of artificial apoptosis induction methods such as cancer radiotherapy, many researchers have attempted to identify the dominant factors of the cell fate decision mechanism.

In this research, IR irradiation is assumed to be the source of DNA damage. IR is frequently used in wet experiments, and DNA double-strand breaks (DSBs) are the most common type of DNA damage induced by IR irradiation. The following biological findings relate to IR-induced cell fate decision. An IR dose of 1 Gy produced 20–40 DSBs per cell, and the distribution of DSB generation followed a Poisson distribution [5]. DSBs generated by IR irradiation were classified into simple DSB (sDSB) and complex DSB (cDSB), with the condition that the incidences of sDSB and cDSB were 60%–80% and 20%–40%, respectively [6]. Regarding the p53 signaling network, ataxia telangiectasia mutated (ATM), checkpoint kinase 2 (Chk2), mouse double minute 2 homolog (Mdm2) and wild-type p53-induced phosphatase (Wip1) were identified as key intranuclear components that generate sustained oscillation of the p53 level (p53 pulse) [7]–[9]. The p53 oscillation was observed at the single-cell level in human breast cancer epithelial MCF7 cells [7], [8]. In IR-sensitive cell lines such as thymus and spleen, oscillatory behavior of p53 was not observed, and the p53 was translocated into mitochondria during 30 minutes after IR-irradiation and directly induced apoptosis [10]. In this study, we focused on the relationship between p53 oscillation and apoptosis induction. The mean amplitude and width of each p53 pulse was constant regardless of IR dose [7]. On the other hand, individual cells exposed to the same IR dose exhibited difference in the number of p53 pulses (p53 dynamic variability), and the number of p53 pulses at the single-cell level tended to increase with the IR dose [7], [8]. In contrast, damped oscillation of the p53 level was observed in cell populations of mouse fibroblasts (NIH3T3 cells) and MCF7 cells in response to IR irradiation, and the amplitudes of oscillations increased with the IR dose [11]. Such oscillations of the p53 level were also observed in mice in vivo, which indicated that oscillations of the p53 level are a general phenomenon in various cell types in higher organisms [12]. An increase in the IR dose effected a change in the fractions of cells that were classified by the number of p53 pulses (the effect of IR intensity on p53 dynamic variability) [7]. Although the apoptosis induction rate in a cell cluster of fibrosarcoma cells increased with IR intensity, differences were observed in intrinsic apoptosis induction at the single-cell level (intrinsic apoptosis induction variability) [4]. These biological findings imply that apoptosis induction at the single-cell level depends on the stochastic behaviors of intranuclear biological reaction processes generated in the p53 signaling network, including DSB generation and repair (intrinsic noise).

*In silico* experiments using mathematical modeling and mathematical analysis are one available method of understanding the cell fate decision mechanism as a result of fluctuations of those cellular responses, i.e., cell-to-cell variability in p53 pulses and apoptosis induction under conditions of various stress intensities. Several mathematical models have been used to explore the mechanism by which the dynamics of p53 affect cell cycle arrest and apoptosis induction [13]–[16]. Till date, one prominent finding was reported by Zhang et al.; they constructed an integrative model of four modules—generation and repair of IR-induced DSBs module, ATM switch module, p53-Mdm2 oscillator module, and cell fate decision module—and reported the possibility that stochasticity in DSB generation led to cell-to-cell variability in cell fate [14]. However, Zhang's model did not take into consideration any stochasticity in the generation of the p53 pulse, and the question of whether any effect of IR dose is observed on the dynamics of the p53 signaling network remained unanswered. The cell fate decision mechanism consists of several signal transduction systems that extend into two spaces, the nucleus and cytoplasm. In general, the existing probability of intranuclear proteins is much smaller than that of cytoplasmic proteins [17]. Hence, the intranuclear biochemical reaction processes develop notable fluctuations compared with the cytoplasmic ones. We inferred that stochasticity in the dynamics of the p53 signaling network also has a profound relationship with cell-to-cell variability in cell fate. This hypothesis is in agreement with the implication based on the abovementioned biological findings [4]–[9], [11], [12]. It is well known that stochastic simulation is useful for exploring the emergence and collapse of biological functions [18]. Our novel mathematical model, which realized stochasticity in both the generation and repair of DSB and the p53 signaling network, has the potential to elucidate the dynamic behavior of the cell fate decision mechanism under conditions of various stress intensities.

In this paper, we describe the construction of a massive integrative model (proposed model) that consists of the generation of IR-induced DSB, DSB repair system, p53 signaling network, and apoptosis induction pathway. These, except for the apoptosis induction pathway, are described as intranuclear biochemical reactions. Because they are modeled as stochastic processes, intrinsic noise is introduced into the simulation of intranuclear reactions. In contrast, the apoptosis induction pathway is described as cytoplasmic reactions and is modeled as a deterministic process. To demonstrate the biological validation of the proposed model, we then compared the abovementioned biological findings observed in NIH3T3 cells, MCF7 cells, and fibrosarcoma cells [4]–[9], [11], [12] with corresponding simulations of the same results. This finding demonstrates the usefulness of the hybrid simulation method, which simultaneously analyzes both the stochastic processes of intranuclear biochemical reactions and the deterministic processes of cytoplasmic biochemical reactions. Finally, using the proposed model, we evaluated the relationship between apoptosis induction and the number of p53 pulses, and we discussed the effects of stochasticity in the generation of IR-induced DSB, DSB repair system, and p53 signaling network on cell fate decision. This paper focused on theoretically demonstrating that the fluctuation of intranuclear biochemical reaction processes in cells is a major factor creating unknown criteria of cell fate decisions.

## Materials and Methods

**Figure 1:**
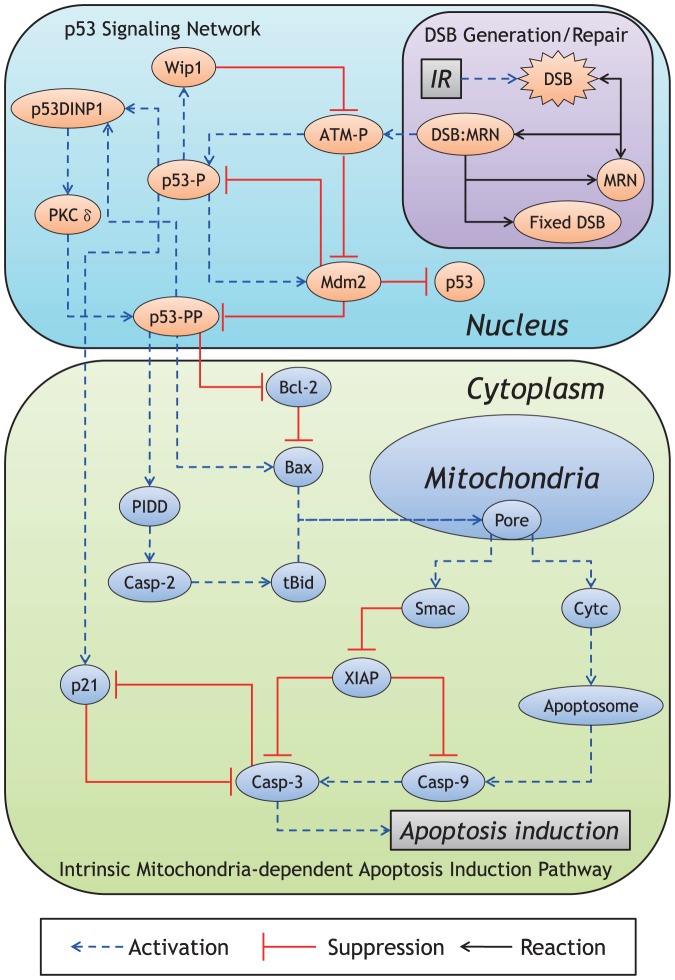
Overview of the proposed model. The proposed model consists of the generation of IR-induced DSB, DSB repair system, and p53 signaling network as nuclear reactions and the apoptosis induction pathway as cytoplasmic reactions. Further, the reactions in the nucleus and cytoplasm are simulated as stochastic and deterministic processes, respectively (see Text S1 *“Direct Hybrid Method”* for details).

**Figure 2:**
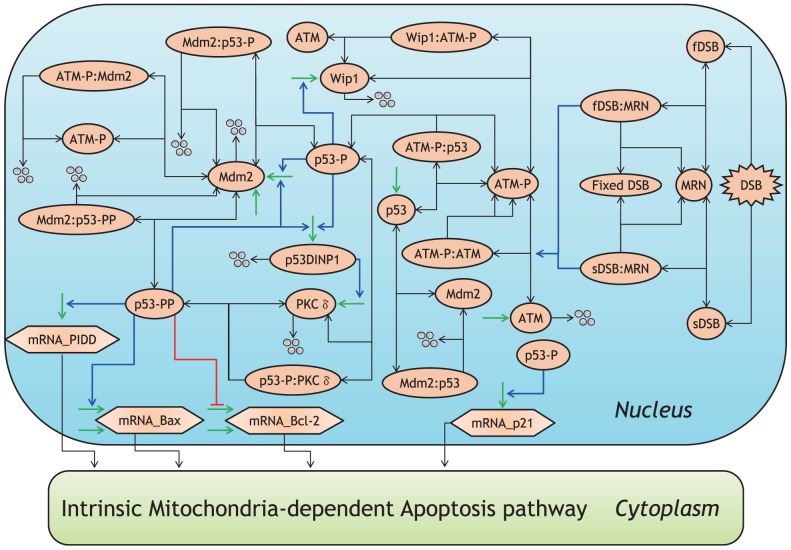
Molecular details of the DSB repair system and p53 signaling network in the nucleus. Activation is represented by blue lines, suppression by red, synthesis by green, degradation by four circles, and binding by black lines. The abbreviations for each molecular species are described in Table S2.

**Figure 3:**
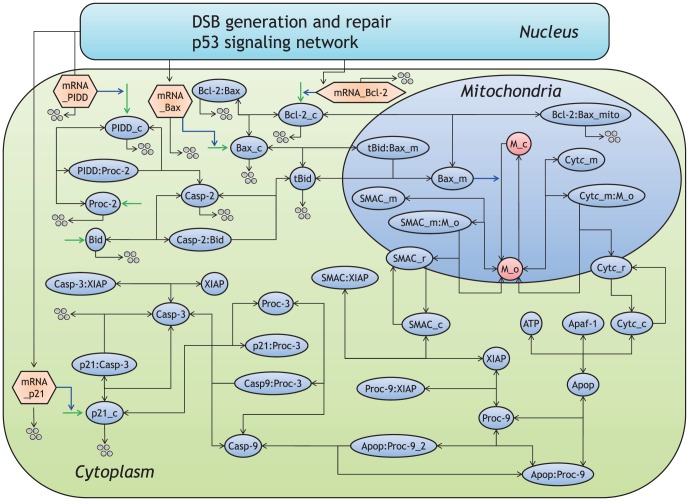
Molecular details of the apoptosis induction pathway in the cytoplasm. Activation is represented by blue lines, suppression by red, synthesis by green, degradation by four circles, and binding by black lines. The abbreviations for each molecular species are described in Table S4.

An overview of the proposed model is shown in Figure 1. Since the existing probability of intranuclear proteins is much smaller than that of cytoplasmic proteins [17], the intranuclear biochemical reaction processes develop notable fluctuations (mesoscopic protein dynamics) compared with the cytoplasmic ones. In the proposed model, the generation of IR-induced DSBs, DSB repair system, and p53 signaling network are described as the intranuclear biochemical reactions (Figure 2), whereas the intrinsic apoptosis induction pathway is described as only cytoplasmic biochemical reactions (Figure 3). The intranuclear and cytoplasmic reactions were simulated as stochastic and deterministic processes (hybrid simulation), respectively. Tables S1, S2, S3, S4, S5 show the kinetic parameter values, the initial conditions of dependent variables and the set of components in the stochastic and deterministic models. The relationship of apoptosis induction with the number of p53 pulses was explored by subjecting the results of the hybrid simulations using the proposed model to mathematical and statistical analyses. Essential features of biological reaction processes in each module are summarized as follows:

### IR-induced DSB generation system

The process of DSB generation was constructed based on the mathematical model proposed by Ma et al. [15]. An IR dose of 1 Gy produced 20–40 DSBs per cell, which were scattered in the Poisson distribution [5]. In DSB generation, the proportion of sDSB to the initial number of DSBs (total DSBs) was 60%–80%, whereas the proportion of cDSB was 20%–40% [6]. We analyzed the distribution of DSB using following proportions: (1) the proportions of sDSB and cDSB were fixed to 70% and 30%. (2) The proportion of sDSB was normal random number whose mean value is 70%. (3) The proportion of sDSB was uniform random number between 60% and 80%. As a result, the distribution of each DSB with fixed proportion showed little discrepancies with those with other proportions (Figures S1, S2, S3). Hence, the proportions of sDSB and cDSB in the proposed model were fixed to 70% and 30%, respectively. Based on these biological findings and our estimations, the number of IR-induced DSBs was calculated in the proposed model using the following procedure: (1) a pulsed IR dose of x Gy was given at 0 h. The value of x was set to 0, 0.3, 2.5, or 6. (2) The total number of DSBs was generated from the Poisson distribution with a mean value of 35×. (3) These DSBs were separated into sDSB and cDSB, with 70% modeled as sDSBs and the remainder modeled as cDSBs.

### DSB repair system

The DSB repair system, similar to DSB generation, was constructed based on the mathematical model proposed by Ma et al. [15]. IR-induced DSBs bound to Mre11-Rad50-Nbs1 (MRN) to form the DSB–MRN complex and were repaired by various repair proteins (Figure 2). When DSB repair was successful, this complex dissociated into repaired DSB (as shown in Figure 2) and the free MRN complex. However, when DSB repair failed, the DSB–MRN complex dissociated into unrepaired DSB (sDSB or cDSB) and the free MRN complex (Figure 2). sDSBs and cDSBs were repaired using the same processes, but the efficiency of sDSB repair was 6–40 times higher than that of cDSB repair [6], [19]. In our simulations, all kinetic parameters for sDSB repair were set to be equal to 40 times those for cDSB repair (details are shown in Table S1).

### p53 signaling network

The reaction scheme of the p53 signaling network is also shown in Figure 2. ATM was autophosphorylated and became active ATM (ATM-P) in the presence of the DSB–MRN complex [20]. In our model, the DSB–MRN complex activated both binding and dissociation of ATM-P and ATM. ATM-P phosphorylated Ser-15 of p53 (p53-P) and promoted the degradation of Mdm2. In turn, p53-P increased the levels of Mdm2, Wip1 and p53-dependent damage-inducible nuclear protein 1 (p53DINP1) in the nucleus and induced the synthesis of p21 mRNA. Wip1 dephosphorylated ATM-P to ATM, causing its inactivation. This formed a negative feedback loop between ATM and p53 [9], [21]. The p53DINP1 activated a kinase, such as protein kinase C δ (PKCδ), that phosphorylated Ser-46 of p53-P [22], [23]. Based on these findings, we assumed that p53DINP1 activated PKCδ in the proposed model (Figure 2). When Ser-46 of p53-P (p53-PP) was phosphorylated by PKCδ, it increased the level of Mdm2 and p53DINP1 in the same way as observed following phosphorylation of p53. In addition, p53-PP induced the synthesis of mRNA of Bax [24] and a p53-induced protein with a death domain (PIDD) and simultaneously suppressed the synthesis of Bcl-2 mRNA [24]. Mdm2 ubiquitinated three forms of p53 (p53, p53-P, and p53-PP) and promoted their degradation. In the proposed model, the mRNA of several apoptosis-related proteins, such as p21, Bax, Bcl-2, and PIDD, were synthesized in the nucleus and rapidly transported from the nucleus to the cytoplasm. We estimated the synthesis rate of chemical species based on the translation rate of four amino acids per second [25]. In addition, the degradation rate of mRNA was set equal to the half-life of each mRNA [26] (details are shown in Table S1).

### Intrinsic apoptosis induction pathway

In the proposed model, the intrinsic apoptosis induction pathway, also known as the mitochondria-dependent intrinsic pathway, was activated by DNA damage, specifically IR-induced DSBs, via the p53 signaling network [27]. The reaction scheme of this pathway is shown in Figure 3. After DSBs were generated in the nucleus, p53-P induced the synthesis of p21 mRNA, and p53-PP induced the mRNA of both Bax and PIDD (Figure 2). The resulting mRNAs of p21, Bax, and PIDD were rapidly transported from the nucleus to the cytoplasm, and the proteins p21_c, Bax_c and PIDD_c were synthesized from their respective mRNAs (Figure 3). Cytoplasmic PIDD_c cleaved procaspase (Proc)-2 to active Casp-2, and Casp-2 subsequently cleaved BH3 interacting domain death agonist (Bid) to active truncated Bid (tBid). tBid then bound to cytoplasmic Bax_c, which was transported from the cytoplasm to the mitochondria. Mitochondrial Bax (Bax_m in Figure 3) enhanced mitochondrial outer membrane permeability (MOMP), resulting in the release of both mitochondrial Cytc and second mitochondria-derived activator of caspase (SMAC) to the cytoplasm [28]–[30]. In the proposed model, Bax_m induced the transition from a state of low MOMP (M_c in Figure 3) to one of high MOMP (M_o in Figure 3). This mediated mitochondrial the release of Cytc_m and SMAC_m into the cytoplasm. The kinetic model of the series of processes necessary for the release of Cytc_m and SMAC_m was constructed based on the mathematical model proposed by Albeck et al. [31]. In the cytoplasm, seven Cytc_c molecules and seven apoptotic protease-activating factor-1 (Apaf-1) molecules formed an apoptosome in an ATP-dependent process [28]. Cytoplasmic Cytc triggered the activation of the caspase cascade in the apoptosis induction pathway [32]. In the proposed model, cytoplasmic Cytc_c simply bound to Apaf-1 and ATP to form an apoptosome (Apop in Figure 3). The apoptosome bound to two molecules of inactive Proc-9 to produce a molecule of active Casp-9 [28], which then cleaved inactive Proc-3 to generate active Casp-3. Both Proc-9 and Casp-3 were inhibited by binding of X-linked inhibitor of apoptosis protein (XIAP) [33]. However, XIAP was trapped by cytoplasmic SMAC_c, which led to the upregulation of Casp-3 [34]. Moreover, both Bcl-2 and p21 in the cytoplasm (Bcl-2_c and p21_c in Figure 3) inhibited apoptosis induction. Bcl-2_c bound to cytoplasmic and mitochondrial Bax to suppress apoptosis induction, and p21_c bound to Proc-3 to prevent Casp-9 from cleaving Proc-3 [35]. In contrast, Casp-3 cleaved p21_c and promoted its degradation, resulting in the forced termination of cell cycle arrest [36]. In mathematical analysis, we considered the activation of Casp-3 to be an indication of completion of apoptosis induction [32]; high levels of Casp-3 activity were equated with apoptosis, whereas low levels of Casp-3 activity were equated with cell survival. In our simulations, all kinetic parameters for the intrinsic apoptosis induction pathway were introduced from both Hua's model [37] and Albeck's model [31] (details are shown in Table S1).

### Mathematical analysis

The hybrid simulation was executed based on the method proposed by Ciliberto et al. [17] and Alfonsi et al. [38]. Intranuclear reactions were stochastically analyzed based on propensities of biochemical reaction processes described in Table S3. In contrast, cytoplasmic reactions were analyzed by employing the system of ordinary differential equations detailed in Table S5. Details of this method are shown in the supporting information (Text S1 *“Direct Hybrid Method”*). In this study, the hybrid simulations for a population of 1000 cells were run using an IR dose of 0, 0.3, 2.5, or 6 Gy. The average of the p53 level from 1000 simulations representing 1000 individual cells, frequency distribution of generated p53 pulses, and proportion of intrinsic apoptosis induction were calculated for each IR dose. The dynamics of p53 pulses at the single-cell level and the cell population level resulting from simulations using the proposed model were compared with the biological findings from several studies [4]–[9], [11], [12], and the biological validity of the proposed model was investigated. Thereafter, we evaluated the relationship between apoptosis induction and the number of p53 pulses and explored the variability of cell fate decision.

## Results/Discussion

### Variability of p53 dynamics

**Figure 4:**
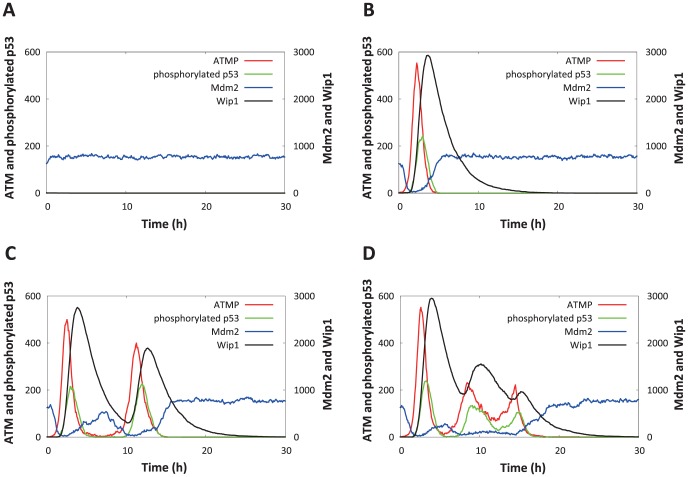
Simulated results of intranuclear species abundance following an IR dose of 2.5 Gy at 0 h. The time courses of phosphorylated p53, Mdm2, ATM-P, and Wip1 in four individual cells with zero (A), one (B), two (C), or three p53 pulses (D) are shown. Each species represents the summation of complexes including the species.

The hybrid simulation, which considered the stochasticity of intranuclear biochemical reaction processes, realized various dynamics of the p53 signaling network. Figure 4 shows a comparison of the time courses of phosphorylated p53, Mdm2, ATM-P, and Wip1 in four individual cells following an IR dose of 2.5 Gy. Here, respective species represented the summation of complexes including the species. During the repair of IR-induced DSBs, the MRN complex and DSB formed the DSB–MRN complex (Figure 2). This activated ATM (ATM-P); ATM-P activated p53 (p53-P) and promoted the degradation of Mdm2, resulting in an increase of total phosphorylated p53. p53-P increased the levels of intranuclear Wip1 and Mdm2. Wip1 then dephosphorylated ATM-P to ATM, while Mdm2 promoted the degradation of p53. Therefore, two negative feedback loops were present between ATM and p53 and between p53 and Mdm2; these negative feedback loops caused several components in the p53 signaling network (e.g., phosphorylated p53, ATM-P, Mdm2, and Wip1) to oscillate. In Figure 4A, the ATM-P level was not elevated following IR irradiation; therefore, p53 located downstream of ATM-P was not phosphorylated. As a result, phosphorylated p53 could not increase, and no p53 pulse was generated. In Figure 4B, ATM-P was elevated following IR irradiation, and a single pulse of ATM-P and phosphorylated p53 was generated. Examples of simulations that generated two and three phosphorylated p53 pulses are shown in Figure 4C and 4D, respectively. Discrepancies in the number of p53 pulses observed in Figure 4 correspond to the variability of p53 dynamics. Moreover, simulations with the IR dose set to 0.3 and 6 Gy are shown in Figures S4 and S5, respectively, in supporting information. This is in qualitative agreement with the simulated results shown in Figure 4.

Despite application of the same IR dose, comparison of the time courses of levels of intranuclear components in cells showed discrepancies in the number of p53 pulses, as shown in Figure 4, Figures S4 and S5. Lahav et al. previously reported that in experiments using the MCF7 cell line, individual cells in the same population generated different numbers of p53 pulses following IR irradiation [7]. Batchelor et al. demonstrated that several components in the p53 signaling network, including p53, ATM-P and Mdm2, exhibited oscillation [9]. Our simulated results based on the proposed model are in good qualitative agreement with these biological findings.

### Effect of IR intensity on variability of p53 dynamics

**Figure 5:**
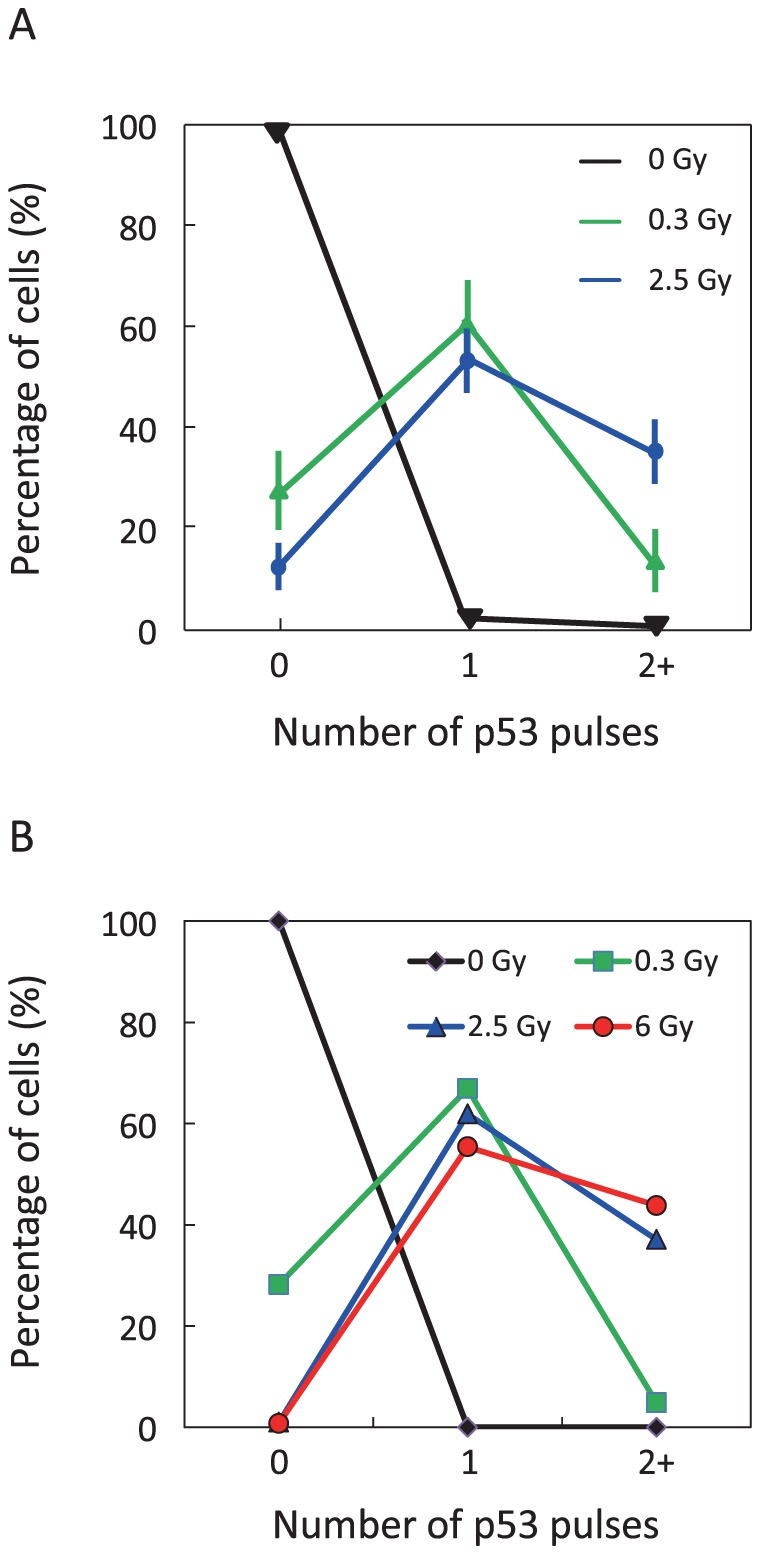
Statistical analysis of p53 pulses. (A) The fraction of cells with zero, one, or two or more p53 pulses as a function of the IR dose is extracted from biological data reported by Lahav et al. [7]. Data are presented as mean ± SEM (B) The fraction of cells with zero, one, or two or more p53 pulses in populations of 1000 cells is calculated from results simulated using different IR doses.

The relationship between IR intensity and the variability of p53 dynamics was explored to confirm the validity of the hybrid simulation based on the proposed model. Following IR irradiation, 1000 cells were classified based on the number of p53 pulses. Figure 5A shows the fractions of MCF7 cells with zero, one, or two or more (2+) p53 pulses as a function of IR dose [7]. Moreover, Figure 5B shows those fractions calculated from the results of the hybrid simulations. In simulations run using an IR dose of 0 Gy, none of the cells showed a p53 pulse. In simulations run using different IR doses, the fractions of cells with zero, one, or 2+ pulses were 28.2%, 66.9%, and 4.9% at 0.3 Gy; 1.0%, 61.9%, and 37.1% at 2.5 Gy; and 0.8%, 55.4%, and 43.8% at 6 Gy, respectively. These simulated results are in quantitative agreement with observed data (Figure 5A and 5B).

Ma et al. indicated that the fluctuations in DSB generation and repair were not enough to reproduce observed proportions of p53 pulses at different IR-dose [15]. They introduced variable kinetic parameters into the model to fit their simulations to experimentally observed data [15]. Though Zhang et al. reproduced variability of p53 pulses by considering only fluctuations in DSB generation and repair, there was no report on which their simulated results were in good agreement with any biological finding [14]. The proposed model with fluctuation in only DSB generation and repair could not realize observed fractions of p53 pulses (Figure S6A). However, our simulations with fluctuations in both DSB generation and repair and intranuclear reactions were quantitatively consistent to experimentally observed data (Figure 5A and 5B). These results showed that the fluctuations in the abundance of both intranuclear chemical species and DSB play an important role as the source of noise.

**Figure 6:**
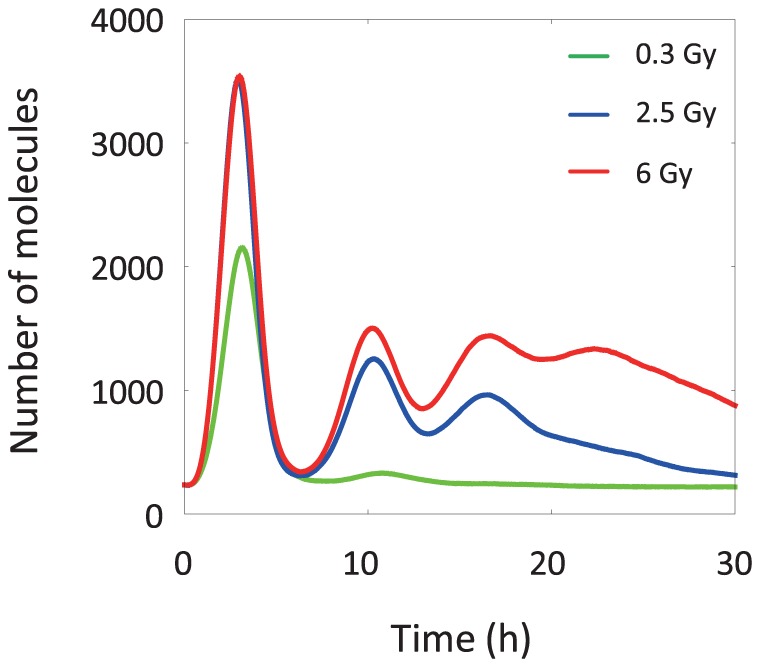
The time courses of total p53 for populations of 1000 cells. Cells are subjected to an IR dose of 0.3, 2.5, or 6 Gy. Total p53 represents the summation of complexes including nuclear p53. p53 oscillations are damped at each IR dose.

Next, the dynamics of p53 pulses at the cell population level were examined. Figure 6 shows the time courses of total p53 for populations of 1000 cells subjected to an IR dose of 0.3, 2.5, or 6 Gy. p53 oscillations were damped at each IR dose. At the cell population level, an increment in the IR dose increased the number of p53 pulses as well as the amplitude of the first p53 pulse. Bar-Or et al. reported that both p53 and Mdm2 exhibited damped oscillation in a population of NIH3T3 cells subjected to IR irradiation, and the amplitude of oscillation was dependent on the IR dose [11]; our simulated results shown in Figure 6 are in agreement with these findings. Taken together, analysis of the simulated results at the single-cell and cell population level demonstrated that damped oscillation of p53 observed in cell populations was produced by the superposition of cell-to-cell variability in the dynamics of p53.

Damped oscillation normally indicates that a biological system is in a relaxed stable state, which is the genesis of the maintenance of homeostasis. In our simulated results, the stochasticity of intranuclear biochemical reaction processes induced heterogeneity and homogeneity of p53 dynamics in single cells and cell populations, respectively. This finding implies that the stochasticity of intranuclear biochemical reaction processes contributes to the implicit spontaneous ordering observed in biological multilayered systems of higher organisms. From the perspective of systems biology, it was reported that stochasticity or fluctuation of biochemical reaction processes enhanced the stability of biological functions [18], [39]. This finding is in good agreement with our findings based on our simulated results. Thus, consideration of the stochasticity of intranuclear biochemical reaction processes was indispensable for enabling the proposed model to realize the variability of p53 dynamics at both the single-cell and cell population levels. The proposed model, which implements such an implicit interlayer regulatory mechanism between cells and tissues, is useful for enhancing the understanding of the dynamic behavior of the cell fate decision mechanism in comparison with other conventional models.

### Variability of intrinsic apoptosis induction

**Figure 7:**
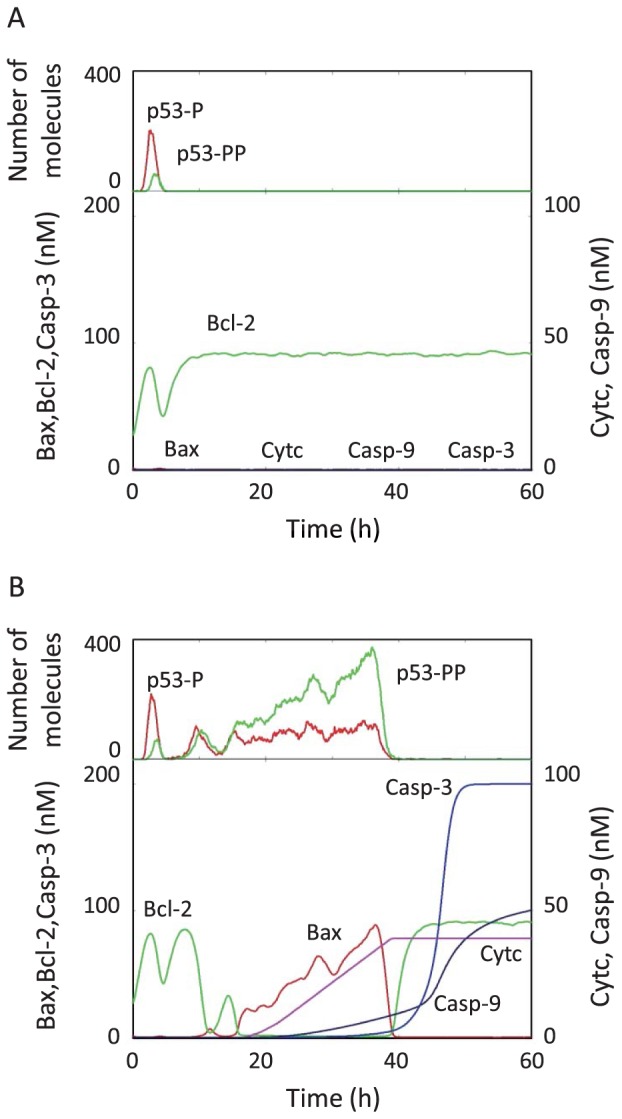
Simulated results of cytoplasmic species following an IR dose of 2.5 Gy at 0 h. The time courses of Bax, Bcl-2, Cytc, Casp-9, Casp-3, p53-P, and p53-PP for two individual cells are shown. Each species represents the summation of complexes including the species. (A) Generation of one p53 pulse did not result in the activation of Casp-3. (B) Generation of multiple p53 pulses resulted in Casp-3 activation.

The effect of the variability of p53 dynamics on downstream cytoplasmic reactions, i.e., the apoptosis induction pathway, was investigated. Figure 7 shows the time courses of Bax, Bcl-2, Cytc, Casp-9, Casp-3, p53-P, and p53-PP following an IR dose of 2.5 Gy. Here, each of these represents the summation of complexes including the species. In the case of generating a single p53 pulse, the concentrations of Bcl-2 and Bax in the cytoplasm were maintained at high and low levels, respectively (Figure 7A). This resulted from the fact that p53-PP, which regulates the synthesis of Bax and Bcl-2, was not increased much in the first p53 pulse. Hence, the species downstream of Bax (e.g., Cytc, Casp-9, and Casp-3) were expressed at low levels, resulting in no apoptosis induction. In contrast, the concentration of Bcl-2 decreased and the concentration of Bax increased when multiple p53 pulses were generated (Figure 7B). A sufficient increment in p53-P in the first p53 pulse induced both p53DINP and PKCδ. These species rapidly increased p53-PP after the second p53 pulse. The increase in Bax and the decrease in Bcl-2 caused the release of Cytc from the mitochondria. Thereby, the concentration of cytoplasmic Cytc increased, and Cytc, Apaf-1, and ATP formed a cytoplasmic apoptosome (Apop in Figure 3). The apoptosome sequentially activated Casp-9 and Casp-3, which ultimately induced apoptosis. These results indicate that the rapid increase of Bax induced by p53-PP was required to induce apoptosis, which is in good agreement with the biological findings [40].

**Figure 8:**
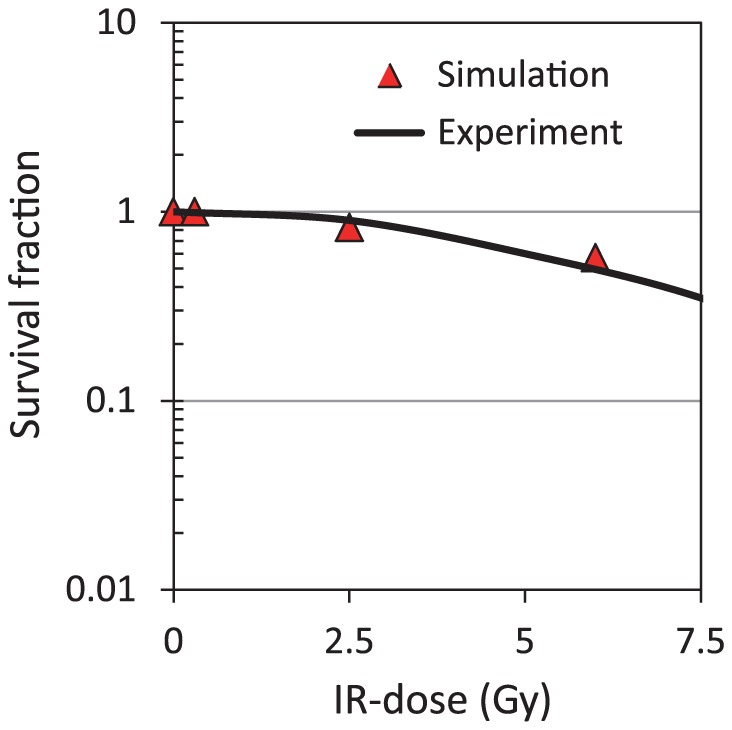
The surviving fraction (SF) of cells in the simulations and experiment data. Simulation (triangle) represented the SF of a population of 1000 cells at IR doses of 0, 0.3, 2.5, and 6 Gy. Experiment (line) indicates the SF of experimental data as observed by Pellegata et al. [4].

Pellegata et al. evaluated the relationship between the apoptosis induction rate and IR intensity [4]. Figure 8 shows a comparison of observed data reported by Pellegata with the simulated results based on our proposed model. Here, triangles represent the fraction of nonapoptotic cells, or the surviving fraction (SF), in a population of 1000 cells following an IR dose of 0, 0.3, 2.5, or 6 Gy in the proposed model, and the SF black line represents the SF of fibrosarcoma cells observed by Pellegata. In Figure 8, increases in the IR dose correlated with decreases in the SF in results generated by the proposed model, and these results are in good agreement with experimental data. Although the simulated results by conventional models showed a tendency of decline in SF with increasing IR-dose [14], [38], there was no comparison of the simulated results with experimentally observed data. In contrast, our simulated results were consistent to observed data in human fibrosarcoma cell line [4], which implied that the proposed model was more quantitative than conventional models. The hybrid simulation based on the proposed model has the potential to realize the variability in intrinsic apoptosis induction.

Next, we explored the relationship between the number of p53 pulses and apoptosis induction. Table 1 lists the number of apoptotic and surviving cells according to the number of p53 pulses and the IR dose. The cells with zero and one p53 pulse all survived, whereas the number of apoptotic cells following treatment with multiple p53 pulses was 0 at 0.3 Gy, 174 at 2.5 Gy, and 429 at 6 Gy. These simulated results based on our proposed model indicate that apoptosis was induced only in cells that had experienced at least two p53 pulses following IR irradiation. In addition, the numerical instability of the part of ordinary differential equations (apoptosis induction pathway) in the proposed model was not detected among 1000 simulations. It suggested that the proposed model was robust within the scope of mathematical analysis in this study.

**Table 1:** The relationship between the number of p53 pulses and apoptosis induction.

IR-dose (Gy)	Zero p53 pulse		One p53 pulse		Two or more p53 pulses	
	Survival	Apoptosis	Survival	Apoptosis	Survival	Apoptosis
0.3	282	0	669	0	49	0
2.5	10	0	619	0	197	174
6	8	0	554	0	9	429
Total	300	0	1842	0	255	603

Populations of 1000 cells at IR-dose of 0.3, 2.5 or 6 Gy were statistically analyzed.

### Cell fate decision

Cellular responses to stressors normally depend on stress intensity and are regulated by the p53 signaling network [2]. Low-intensity stress activates p53 that induces the synthesis of p21; p21 then inhibits cyclin/cyclin-dependent kinase complexes, the cell cycle engines, leading to cell cycle arrest. During this arrest, p53 also activates the DNA repair system. In contrast, high-intensity stress activates p53 that induces apoptosis in addition to cell cycle arrest and DNA repair [3], [16], [41], [42]. p53 induces the intrinsic mitochondria-dependent apoptotic pathway [27]. These biological findings are in qualitative agreement with our simulated results, which showed that a higher IR dose increased the number of p53 pulses as well as the potential for intrinsic mitochondria-dependent apoptosis induction.

**Figure 9:**
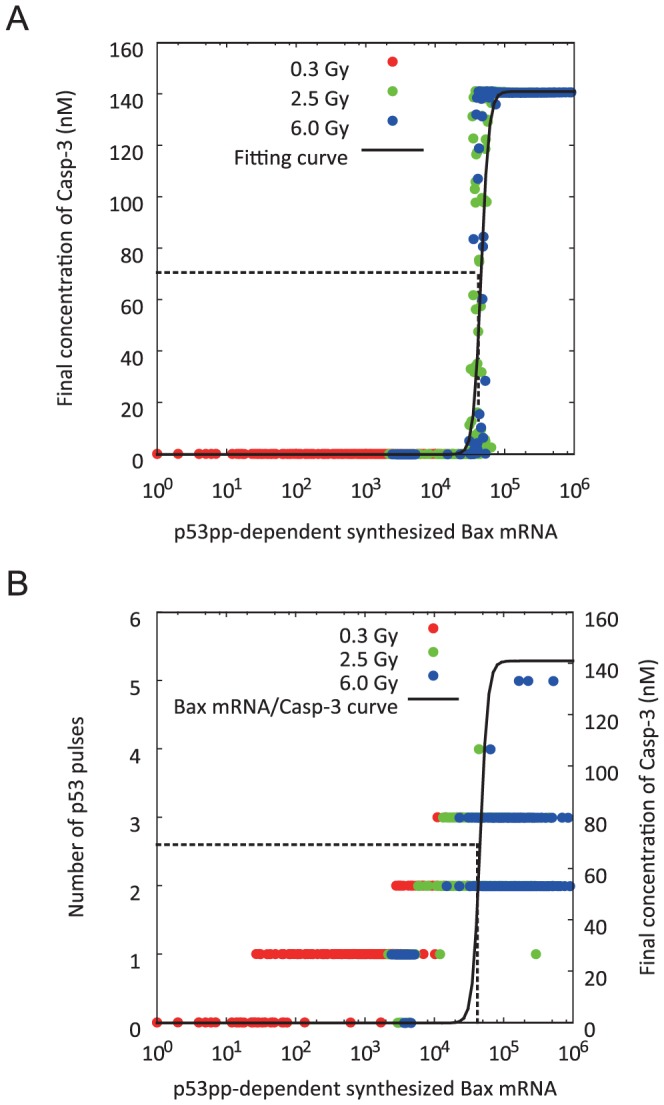
Exploring the dominant factor of the intrinsic mitochondria-dependent apoptosis induction. (A) Relationship between the final concentration of caspase3 and the p53pp-dependent synthesized Bax mRNA abundance. (B) Relationship among the number of p53 pulses, the final concentration of caspase3 and the p53pp-dependent synthesized Bax mRNA abundance. Closed symbol of red, green and blue: IR dose of 0.3, 2.5 and 6.0 Gy, respectively; solid line: regression curve by Hill equation (Hill coefficient = 7.91); broken line: half maximal effective chemical species (Bax) abundance (EC50) to final concentration of caspase3. EC50 was equal to 45816. Caspase3 activation required the generation of multiple p53 pulses. There was individual variability in the number of p53 pulses in cells which were subjected to the same IR dose. The individual variability affected the intrinsic mitochondria-dependent apoptosis induction.

In the early stage of intrinsic apoptosis induction due to DSBs, p53pp activates a synthesis of mRNA of Bax and inactivates that of Bcl-2. Figure 9A shows a relationship between the final concentration of caspase3 and the p53pp-dependent synthesized Bax mRNA abundance, in our simulated results. An analysis based on Hill equation detected the half maximal effective chemical species (Bax) abundance to caspase3 (EC50) of 45815, which indicated that the samples was thinly distributed at EC50. Figure 9B shows a relationship among the number of p53 pulses, the final concentration of caspase3 and the p53pp-dependent synthesized Bax mRNA abundance. The number of p53 pulses at EC50 was equal to 2 or 3. The caspase3 activation required a generation of multiple p53 pulses as shown in Table 1. Thus, it seemed that the intrinsic mitochondria-dependent apoptosis induction was regulated by employing a threshold which was expressed as a function of the p53pp-dependent synthesized Bax mRNA abundance. As observed in Figure 4 and 5 and Table 1, cells, however, exhibited considerable individual variability in p53 dynamics. Loewer et al. had also experimentally observed such considerable individual variability in p53 dynamics [43]. Since the p53pp is final reactant in the p53 signaling network, individual variability in p53 signaling network quantitatively has an impact on both synthetic processes of Bax and Bcl-2. Hence individual variability in the number of p53 pulses affects the intrinsic mitochondria-dependent apoptosis induction (Figure 9B). As a result, our mathematical analysis of the proposed model identified the stochasticity of intranuclear biochemical reaction processes is controlling the final decision of cell fate (mesoscopic protein dynamics).

The artificial regulation of intrinsic apoptosis induction shows promise for novel treatment of tumors. In such studies, a compelling need exists for elucidation of the variability of p53 dynamics in both single cells and cell populations. In this study, our model, which introduced intrinsic noise or stochasticity into the nuclear reactions, was able to more accurately predict criteria of cell fate decision than conventional mathematical models. The simulated results from this study indicate that monitoring the number of p53 pulses following DNA damage may facilitate accurate prediction of the fraction of surviving cells. As a result, it may be possible to estimate the therapeutic efficacy of radiotherapy of cancer cells, which will contribute to the development of better cancer therapies.

### Source of noise

In our proposed model, the process of DSB generation and repair was constructed based on the mathematical model proposed by Ma et al. [15]. Ma's model can statistically imitate a fluctuation in the number of DSBs. Zhang et al. also set the process of DSB generation and repair to the source of noise as well as our proposed model [14]. However, there was no report which assayed a relationship between their simulated data and biological findings. We incorporated not only “the fluctuation in the number of DSBs” but also “the fluctuations in intranuclear reaction rates of the p53 signaling network” as source of noise into our proposed model. With considering both sources of noise, our simulated results were in quantitative agreement with major biological findings observed in human breast cancer epithelial MCF7, NIH3T3, and fibrosarcoma cells [4], [7]–[9], [11], [12]. These findings implied that the stochasticity of intranuclear biochemical reaction processes has a grave influences on the cell fate decision. Hence, we concluded that the fluctuations in the abundance of both intranuclear chemical species and DSB play an important role as the source of noise.

### Limitations

When all reactions were simulated deterministically, the variability of both p53 dynamics and apoptosis induction with IR dose could not be realized (Figure S6). In the case of stochastic simulation, additional longer computation time was required in comparison with the hybrid simulation. Because the frequency of cytoplasmic biochemical reaction processes showed a discrepancy compared with that of intranuclear processes, it was difficult to temporally coordinate intranuclear biochemical reactions with the intrinsic apoptosis induction system (Figure S6). In contrast, the hybrid simulation method more accurately and efficiently realized various biological findings of both the p53 signaling network and the apoptosis induction system. However, the survival fraction curve, shown in Figure 8, varies depending on the cell line used [44]. The proposed model may realize the apoptosis induction rate in other mammalian cell lines by optimizing values of several kinetic parameters and network structures, making it possible to elucidate the cell fate decision mechanism for these other cell lines.

## Conclusions

A massive integrative mathematical model of DSB generation, DSB repair system, p53 signaling network, and apoptosis induction pathway was constructed. The proposed model partitioned reaction processes in a cell into two subsets, intranuclear reactions and cytoplasmic reactions. The intranuclear reactions were modeled as stochastic processes and the cytoplasmic reactions as deterministic processes, and both reaction sets were simulated simultaneously (hybrid simulation). To explore the relationship of cellular responses to stress intensity, cells were subjected to an IR dose of 0, 0.3, 2.5, or 6.0 Gy. The simulated results at the single-cell level showed that the model generated several sustained oscillations of p53, Mdm2, ATM, and Wip1, and cell-to-cell variability in the number of p53 pulses depended on IR intensity. The model also generated damped p53 oscillations in cell populations, and IR intensity affected the amplitudes of the first p53 pulses. Moreover, cells subjected to the same IR dose exhibited variability in apoptosis induction. These simulated results are in good agreement with several biological findings observed in MCF7, NIH3T3, and fibrosarcoma cells, demonstrating that the proposed model was biologically appropriate. Statistical analysis of the simulated results shows that the generation of multiple p53 pulses was a prerequisite for apoptosis induction. Moreover, cells exhibited considerable individual variability in p53 dynamics, which correlated with intrinsic apoptosis induction. The simulated results based on our proposed model demonstrate that the stochasticity of intranuclear biochemical reaction processes controls the final decision of cell fate associated with DNA damage. Applying stochastic simulation to an exploration of intranuclear biochemical reaction processes is indispensable to enhancing the understanding of the dynamic characteristic of biological multilayered systems of higher organisms.

## Supporting Information

Figure S1 Distribution of total DSB (A), simple DSB (B) and complex DSB (C) in different type of proportions of simple DSB at IR-dose of 0.3 Gy. The distribution of total DSB was calculated from 100,000 samples following Poisson distribution with mean value of 35*IR-dose. Simple and complex DSBs were calculated from total DSB. The proportion of simple DSB was fixed to 0.7 (red), normal random number whose mean value is 0.7 (green) or uniform random number between 0.6 and 0.8 (blue).
(PDF)

Figure S2 Distribution of total DSB (A), simple DSB (B) and complex DSB (C) in different type of proportions of simple DSB at IR-dose of 2.5 Gy. The distribution of total DSB was calculated from 100,000 samples following Poisson distribution with mean value of 35*IR-dose. Simple and complex DSBs were calculated from total DSB. The proportion of simple DSB was fixed to 0.7 (red), normal random number whose mean value is 0.7 (green) or uniform random number between 0.6 and 0.8 (blue).
(PDF)

Figure S3 Distribution of total DSB (A), simple DSB (B) and complex DSB (C) in different type of proportions of simple DSB at IR-dose of 6.0 Gy. The distribution of total DSB was calculated from 100,000 samples following Poisson distribution with mean value of 35*IR-dose. Simple and complex DSBs were calculated from total DSB. The proportion of simple DSB was fixed to 0.7 (red), normal random number whose mean value is 0.7 (green) or uniform random number between 0.6 and 0.8 (blue).
(PDF)

Figure S4 Simulated results of nuclear species abundance following an IR-dose of 0.3 Gy at 0 hour. Time courses of phosphorylated p53, Mdm2, ATM-P and Wip1 in four individual cells with No (A), one (B), two (C) and three p53 pulses (D) were shown. Respective species represents the summation of complexes including the species.
(PDF)

Figure S5 Simulated results of nuclear species abundance following an IR-dose of 6.0 Gy at 0 hour. Time courses of phosphorylated p53, Mdm2, ATM-P and Wip1 in four individual cells with No (A), one (B), three (C) and four p53 pulses (D) were shown. Respective species represents the summation of complexes including the species.
(PDF)

Figure S6 Comparison of the simulation results when all reactions were simulated deterministically or stochastically. A hundred cells were simulated by each method, and the results were statistically analyzed. The fraction of cells with zero, one and two or more p53 pulses as a function of IR-dose in only deterministic (A) or stochastic simulations (B) was calculated. The fraction of surviving cells (SF) in the deterministic (C) or stochastic simulations (D) was shown with the Experimental data by Pellegata et al. [4].
(PDF)

Table S1 Kinetic parameters in the proposed model.
(PDF)

Table S2 Initial conditions for each nuclear species.
(PDF)

Table S3 Biochemical reactions in the nucleus.
(PDF)

Table S4 Initial conditions of cytoplasmic species.
(PDF)

Table S5 Ordinary differential equations used for each cytoplasmic reaction.
(PDF)

Text S1 Detailed numerical calculation method.
(PDF)
