# Supplementary material for: Stochasticity of Intranuclear Biochemical Reaction Processes Controls the Final Decision of Cell Fate Associated with DNA Damage
Source: PLoS One. 2014 Jul 8;9(7):e101333. doi: 10.1371/journal.pone.0101333 (PMC4086823; doi:10.1371/journal.pone.0101333)
Supplement: Figure S2 — Distribution of total DSB (A), simple DSB (B) and complex DSB (C) in different type of proportions of simple DSB at IR-dose of 2.5 Gy. The distribution of total DSB was calculated from 100,000 samples following Poisson distribution with mean value of 35*IR-dose. Simple and complex DSBs were calculated from total DSB. The proportion of simple DSB was fixed to 0.7 (red), normal random number whose mean value is 0.7 (green) or uniform random number between 0.6 and 0.8 (blue). (PDF) [file pone.0101333.s002.pdf]

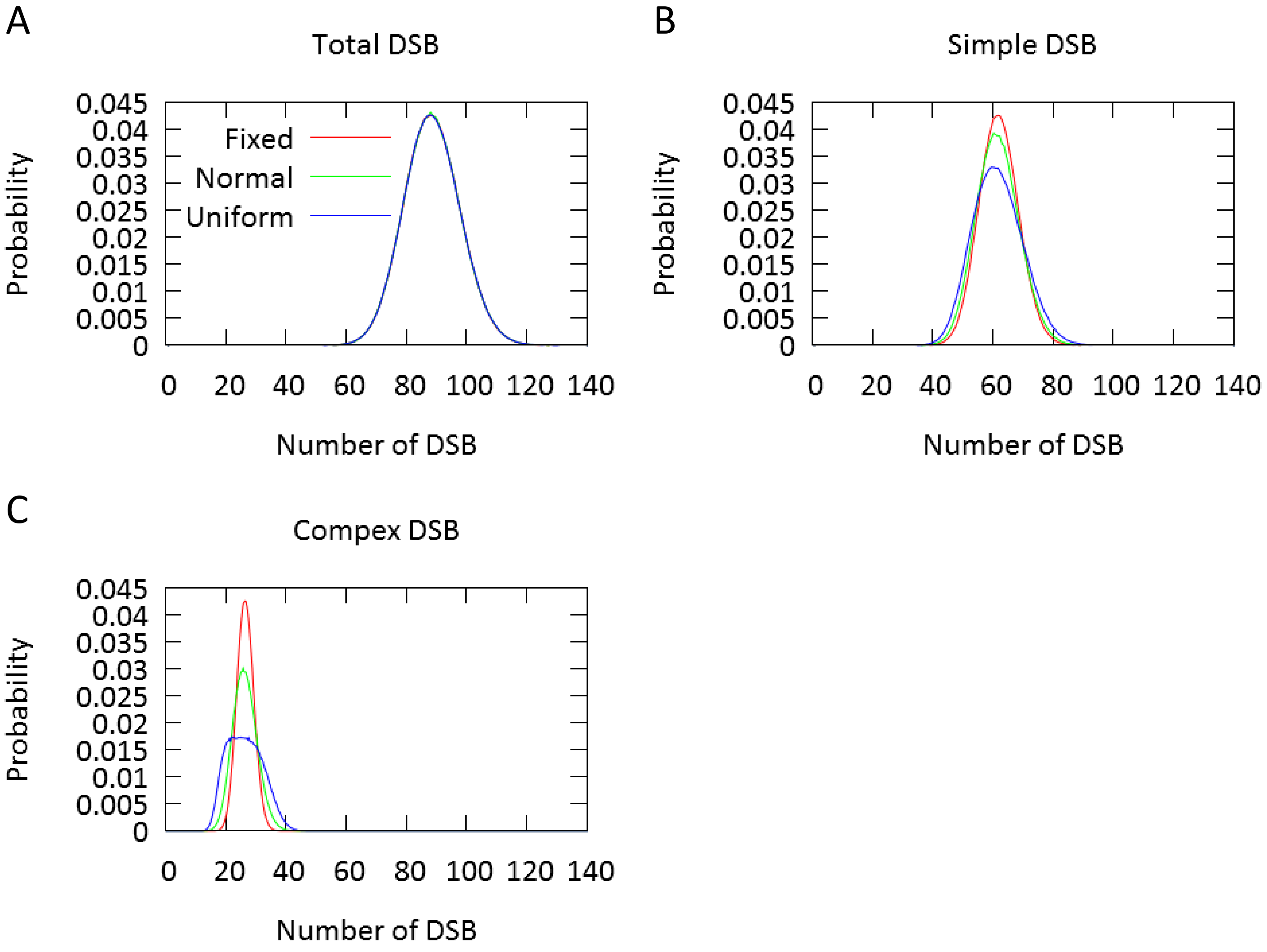

Figure S2 Distribution of total DSB (A), simple DSB (B) and complex DSB (C) in different type of proportions of simple DSB at IR-dose of **2.5 Gy**.

The distribution of total DSB was calculated from 100,000 samples following Poisson distribution with mean value of 35*IR-dose. Simple and complex DSBs were calculated from total DSB. The proportion of simple DSB was fixed to 0.7 (red), normal random number whose mean value is 0.7 (green) or uniform random number between 0.6 and 0.8 (blue).
