# Supplementary material for: Stochasticity of Intranuclear Biochemical Reaction Processes Controls the Final Decision of Cell Fate Associated with DNA Damage
Source: PLoS One. 2014 Jul 8;9(7):e101333. doi: 10.1371/journal.pone.0101333 (PMC4086823; doi:10.1371/journal.pone.0101333)
Supplement: Figure S4 — Simulated results of nuclear species abundance following an IR-dose of 0.3 Gy at 0 hour. Time courses of phosphorylated p53, Mdm2, ATM-P and Wip1 in four individual cells with No (A), one (B), two (C) and three p53 pulses (D) were shown. Respective species represents the summation of complexes including the species. (PDF) [file pone.0101333.s004.pdf]

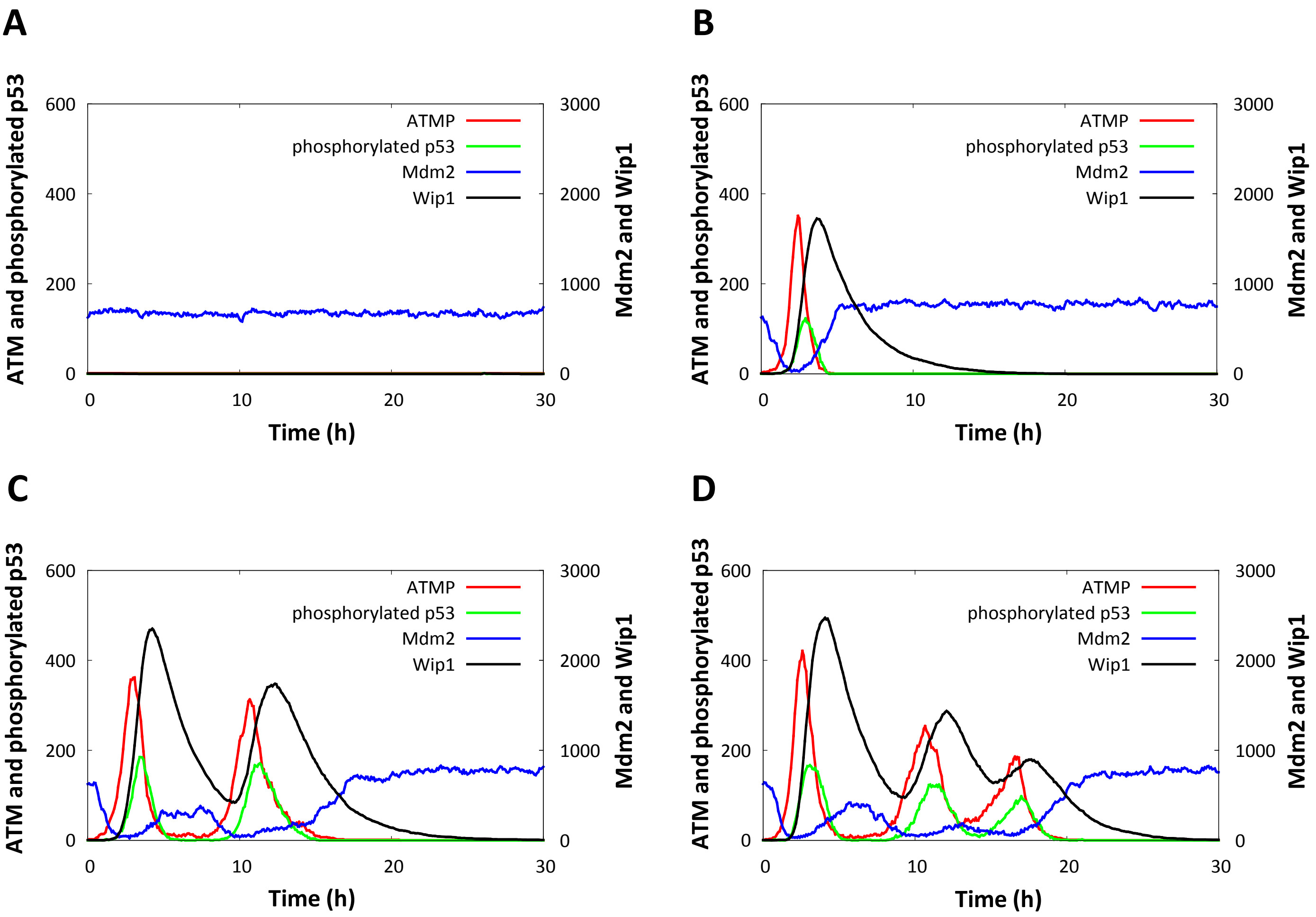

Figure S4 Simulated results of nuclear species following an IR-dose of 0.3 Gy at 0 hour.

Time courses of phosphorylated p53, Mdm2, ATM-P and Wip1 in four individual cells with No (A), one (B), two (C) and three p53 pulses (D) were shown. Respective species represents the summation of complexes including the species.
