# Supplementary material for: Stochasticity of Intranuclear Biochemical Reaction Processes Controls the Final Decision of Cell Fate Associated with DNA Damage
Source: PLoS One. 2014 Jul 8;9(7):e101333. doi: 10.1371/journal.pone.0101333 (PMC4086823; doi:10.1371/journal.pone.0101333)
Supplement: Figure S6 — Comparison of the simulation results when all reactions were simulated deterministically or stochastically. A hundred cells were simulated by each method, and the results were statistically analyzed. The fraction of cells with zero, one and two or more p53 pulses as a function of IR-dose in only deterministic (A) or stochastic simulations (B) was calculated. The fraction of surviving cells (SF) in the deterministic (C) or stochastic simulations (D) was shown with the Experimental data by Pellegata et al. [4]. (PDF) [file pone.0101333.s006.pdf]

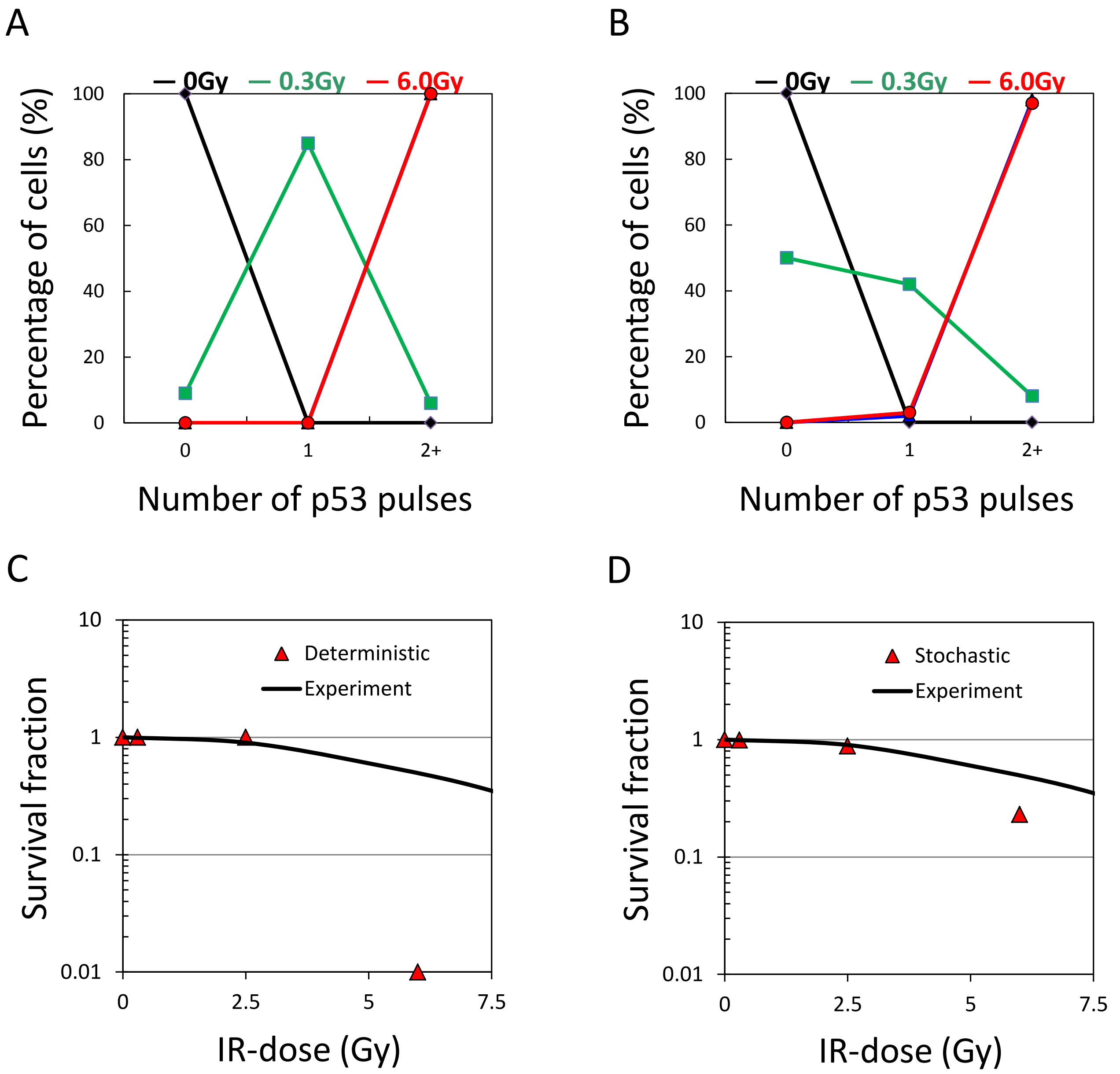

Figure S6 Comparison of the simulation results when all reactions were simulated deterministically or stochastically.

A hundred cells were simulated by each method, and the results were statistically analyzed. The fraction of cells with zero, one and two or more p53 pulses as a function of IR-dose in only deterministic (A) or stochastic simulations (B) was calculated. The fraction of surviving cells (SF) in the deterministic (C) or stochastic simulations (D) was shown with the Experimental data by Pellegata et al. [1].

Reference:

[1] Pellegata NS, Antoniono RJ, Redpath JL, Stanbridge EJ (1999) DNA damage and p53-mediated cell cycle arrest: A reevaluation. Proc Natl Acad Sci USA 93: 15209–15214.
