# Supplementary material for: Stochasticity of Intranuclear Biochemical Reaction Processes Controls the Final Decision of Cell Fate Associated with DNA Damage
Source: PLoS One. 2014 Jul 8;9(7):e101333. doi: 10.1371/journal.pone.0101333 (PMC4086823; doi:10.1371/journal.pone.0101333)
Supplement: Table S1 — Kinetic parameters in the proposed model. (PDF) [file pone.0101333.s007.pdf]

Table S1 Kinetic parameters in the proposed model

Kinetic parameter	Value	Description	References
s1	7.83E-01	Rate of p53 synthesis	393AA, 98.25sec *1
s2	6.60E-03	Rate of Wip1 synthesis by p53-P	605AA, 151sec *1
s3	8.10E-03	Rate of Mdm2 synthesis by p53-P and p53-PP	491AA, 122sec *1
s3d	6.34E-01	Basal rate of Mdm2 synthesis	Estimated
s4	1.02E-1	Basal rate of ATM synthesis	3066AA, 766sec *1
s5	1.67E-02	Rate of p53DINP1 by p53-P and p53-PP	
s6	5.91E-03	Rate of PKCδ activation by p53-P	
s7	1.31	Basal rate of Bcl-2 mRNA synthesis	
s7d	1.67E-02	Inhibition rate of Bcl-2 mRNA synthesis by p53-PP	
s8	1.43	Basal rate of Bax mRNA synthesis	
s8d	1.83E-02	Rate of Bax mRNA synthesis by p53-PP	
s9	4.37E-03	Rate of PIDD mRNA by p53-PP	
s10	2.44E-02	Rate of p21 mRNA by p53-P	
d2	1.30E-04	Degradation rate of Wip1	
d3	8.30E-04	Degradation rate of Mdm2	
d4	1.00E-04	Degradation rate of ATM	
d5	2.00E-02	Degradation rate of p53DINP1	
d6	1.00E-05	Degradation rate of PKCδ	
f0	1.28E-6	Binding rate of ATM-P and Wip1	
r0	1	Dissociation rate of ATM-P and Wip1	Estimated
k0	1.6	Dephosphorylation rate of ATM-P by Wip1	
f1	1.28E-06	Binding rate of ATM-P and p53	
r1	1.00E-02	Dissociation rate of ATM-P and p53	
k1	1.00E-03	Phosphorylation rate of p53 by ATM-P	
f2	1.20E-04	Binding rate of p53 and Mdm2	
r2	2.06	Dissociation rate of p53 and Mdm2	
k2	1.00E-01	Ubiquitination rate of p53 by Mdm2	
f2p	9.83E-05	Binding rate of p53-P and Mdm2	
r2p	2	Dissociation rate of p53-P and Mdm2	
k2p	1.00E-01	Ubiquitination rate of p53-P by Mdm2	
f3	1.28E-04	Binding rate of ATM-P and Mdm2	
r3	1.00E-01	Dissociation rate of ATM-P and Mdm2	
k3	1.00E-01	Phosphorylation rate of Mdm2 by ATM-P	
f4	1.28E-06	Binding rate of ATM and ATM-P	
r4	1.01	Dissociation rate of ATM and ATM-P	

k4	1.01	Phosphorylation rate of ATM by ATM-P	Estimated
f6	9.83E-05	Binding rate of p53-PP and Mdm2	
r6	2	Dissociation rate of p53-PP and Mdm2	
k6	1.00E-01	Ubiquitination rate of p53-P by Mdm2	
kmt	4.16E-03	Transport rate from nucleus to cytoplasm	[2]
s_bind	1.32E-01	Binding rate of s_DSB and MRN complex	[3]
s_dis	3.24E-02	Dissociation rate of s_DSB and MRN complex	
s_rep	2.04E-03	Repair rate of s_DSB	
c_bind	3.30E-03	Binding rate of c_DSB and MRN	
c_dis	8.10E-04	Dissociation rate of c_DSB and MRN complex	
c_rep	5.10E-05	Repair rate of c_DSB	
s_pro2	8.85E-03	Basal rate of procaspase-2 synthesis	452AA, 113sec *1
s_bid	2.05E-02	Basal rate of Bid synthesis	195AA, 48sec *1
s_bax	1.83E-2	Rate of Bax synthesis	218AA, 54sec *1
s_bcl	1.67E-2	Rate of Bcl-2 synthesis	239AA, 59sec *1
s_pidd	4.37E-3	Rate of PIDD synthesis	915AA, 228sec *1
s_p21	2.44E-2	Rate of p21 synthesis	164AA, 41sec *1
d_pro2	3.00E-04	Degradation rate of Proc-2 and Casp-2	Estimated
d_bax	1.20E-02	Degradation rate of Bax	[4]
d_bcl	2.44E-04	Degradation rate of Bcl-2	
d_bid	8.20E-04	Degradation rate of Bid	
d_pidd	1.00E-03	Degradation rate of PIDD	Estimated
d_p21	1.00E-04	Degradation rate of p21	Estimated
d_mbax	9.92E-5	Degradation rate of Bax mRNA	Estimated
d_mbcl	6.17E-4	Degradation rate of Bcl-2 mRNA	*2
d_mp21	5.56E-4	Degradation rate of p21 mRNA	*2
d_mpidd	7.00E-4	Degradation rate of PIDD mRNA	Estimated
k1f	2.00E-04	Binding rate of Bax and tBid	[4]
k1r	2.00E-02	Dissociation rate of Bax and tBid	
k1c	5.00E-03	Transport rate of Bax/tBid into mitochondria	
k4f	7.00E-03	Binding rate of SMAC and XIAP	
k4r	2.21E-03	Dissociation rate of SMAC and XIAP	
k5f	2.78E-07	Binding rate of Cytc, Apaf-1 and ATP	
k5r	5.70E-03	Dissociation rate of Cytc, Apaf-1 and ATP	
k6f	2.84E-04	Binding rate of apoptosome and Proc-9	
k6r	7.49E-02	Dissociation rate of apoptosome and Proc-9	
k7f	4.41E-04	Binding rate of apoptosome and Proc-9	
k7r	1.00E-01	Dissociation rate of apoptosome and Proc-9	

k8	7.00E-01	Cleaving rate of Proc-9 by apoptosome	
k9f	1.96E-05	Binding rate of Casp-9 and Proc-3	
k9r	5.71E-02	Dissociation rate of Casp-9 and Proc-3	
k10	4.8	Cleaving rate of Proc-3 by Casp-9	
k11f	1.06E-04	Binding rate of Proc-9 and XIAP	
k11r	1.00E-03	Dissociation rate of Proc-9 and XIAP	[4]
k12f	2.50E-03	Binding rate of Casp-3 and XIAP	
k12r	2.40E-03	Dissociation rate of Casp-3 and XIAP	
k13f	2.00E-03	Binding rate of Bax and Bcl-2	
k13r	2.00E-02	Dissociation rate of Bax and Bcl-2	
k14f	5.00E-04	Binding rate of PIDD and Proc-2	Estimated
k14r	2.00E-01	Dissociation rate of PIDD and Proc-2	Estimated
k15	1.00E-01	Activation rate of Proc-2 by PIDD	Estimated
k16f	5.00E-03	Binding rate of Bid and Casp-2	
k16r	5.00E-03	Dissociation rate of Bid and Casp-2	[5] *3
k17	1.00E-01	Cleaving rate of Bid by Casp-2	
k18f	6.00E-04	Binding rate of p21 and Proc-3 or Casp-3	Estimated
k18r	1.00E-03	Dissociation rate of p21 and Proc-3 or Casp-3	Estimated
k19	1	Cleaving rate of p21 by Casp-3	Estimated
mf1	1.20E-03	Binding rate of SMAC or Cytc and mitochondrial pore	
mr1	1.00E-02	Dissociation rate of SMAC or Cytc and mitochondrial pore	
mk1	1.00E-03	Release rate of SMAC and Cytc	[5]
mk2	1.00E-03	Transport rate of SMAC and Cytc between mitochondria and cytoplasm	
mf3	1.00E-03	Penetration rate of mitochondrial membrane by Bax	Estimated
mJ3	5		Estimated
mr3	20	Recovery rate of mitochondrial membrane	Estimated

*1 We assumed the synthesis rate based on the translation rate of four amino acids per second [1].

*2 We assumed the degradation rate based on the half-life of each mRNA [2].

*3 We assumed that the binding/dissociation/ cleaving rates of Bid and Casp-2 was as same as those of Bid and Casp-8.

Reference:

[1] Audibert, A, Weil, D, Dautry, F. (2002) In Vivo Kinetics of mRNA Splicing and Transport in Mammalian Cells. *Mol. Cell Biol* 22(19): 6706-6718.

[2] Kren, BT, Steer, CJ, (1996) Posttranscriptional regulation of gene expression in liver regeneration: role of mRNA stability. *EMBO J* 10:819-828.

[3] Ma L, et al. (2005) A plausible model for the digital response of p53 to DNA damage. *Proc Natl Acad Sci USA* 102: 14266–14271.

[4] Hua F, Cornejo MG, Cardone MH, Stokes CL, Lauffenburger DA (2005) Effects of Bcl-2 levels on Fas signaling-induced caspase-3 activation: molecular genetic tests of computational model predictions. *J Immunol* 75(2): 985-995.

[5] Albeck JG, Burke JM, Spencer SL, Lauffenburger, DA, Sorger PK (2008) Modeling a snap-action, variable-delay switch controlling extrinsic cell death. *PLoS Biol* 6: e299.
