# Supplementary material for: Stochasticity of Intranuclear Biochemical Reaction Processes Controls the Final Decision of Cell Fate Associated with DNA Damage
Source: PLoS One. 2014 Jul 8;9(7):e101333. doi: 10.1371/journal.pone.0101333 (PMC4086823; doi:10.1371/journal.pone.0101333)
Supplement: Table S2 — Initial conditions for each nuclear species. (PDF) [file pone.0101333.s008.pdf]

Table S2 Initial conditions for each nuclear species

Species	Value (N)	Description
ATM	1017	Inactive ataxia telangiectasia mutated.
ATM:ATM-P	0	Complex of ATM with ATM-P
ATM-P	1	Active ATM
ATM-P:Wip1	0	Complex of ATM-P with Wip1
cBax_mRNA	*1	Bax mRNA in cytoplasm
nBax_mRNA	35	Bax mRNA in nucleus
cBcl-2_mRNA	*1	Bcl-2 mRNA in cytoplasm
nBcl-2_mRNA	300	Bcl-2 mRNA in nucleus
Mdm2	626	Murine double minute 2
Mdm2:ATM-P	0	Complex of Mdm2 with ATM-P
MRN	40	Mre11-Rad50-Nbs1 complex
cp21_mRNA	*1	p21 mRNA in cytoplasm
np21_mRNA	0	p21 mRNA in nucleus
p53	235	Inactive p53
p53:ATM-P	0	Complex of p53 with ATM-P
p53:Mdm2	0	Complex of p53 with Mdm2
p53DINP1	0	p53-dependent damage-inducible nuclear protein 1
p53-P	0	Active p53 whose Ser15 was phosphorylated
p53-P:Mdm2	0	Complex of p53-P with Mdm2
p53-PP	0	Active p53 whose Ser15 and Ser46 were phosphorylated
p53-PP:Mdm2	0	Complex of p53-PP with Mdm2
cPIDD_mRNA	*1	p53-induced protein with a death domain (PIDD) mRNA in cytoplasm
nPIDD_mRNA	0	PIDD mRNA in nucleus
PKCδ	0	Protein kinase C δ
PKCδ:p53-P	0	Complex of PKCδ with p53-P
Wip1	4	Wild-type p53-induced phosphatase 1

*1 Calculated using the concentration of cytoplasmic species (see conversion of concentration into the number of molecules)
