# Supplementary material for: Stochasticity of Intranuclear Biochemical Reaction Processes Controls the Final Decision of Cell Fate Associated with DNA Damage
Source: PLoS One. 2014 Jul 8;9(7):e101333. doi: 10.1371/journal.pone.0101333 (PMC4086823; doi:10.1371/journal.pone.0101333)
Supplement: Table S3 — Biochemical reactions in the nucleus. (PDF) [file pone.0101333.s009.pdf]

Table S3 Biochemical reactions in the nucleus

No.	Propensity function	Reaction
1	s1	p53 synthesis
2	s2*p53-P	Wip1 synthesis induced by p53-P
3	s3*(p53-P+p53-PP)	Mdm2 synthesis induced by p53-P and p53-PP
4	s3d	Mdm2 synthesis
5	s4	ATM synthesis
6	s5*(p53-P+p53-PP)	p53DINP1 synthesis induced by p53-P and p53-PP
7	s6*p53DINP1	PKCδ synthesis induced by p53DINP1
8	s7	Bcl-2_mRNA synthesis
9	s7d*p53-PP	Inhibition of Bcl-2_mRNA synthesis by p53-PP
10	s8	Bax_mRNA synthesis
11	s8d*p53-PP	Bax_mRNA synthesis induced by p53-PP
12	s9*p53-PP	PIDD_mRNA synthesis induced by p53-PP
13	s10*p53-P	p21_mRNA synthesis induced by p53-P
14	d2*Wip1	Wip1 degradation
15	d3*Mdm2	Mdm2 degradation
16	d4*ATM	ATM degradation
17	d5*p53DINP1	p53DINP1 degradation
18	d6*PKCδ	PKCδ degradation
19	kmt*Bcl-2_mRNA	Bcl-2_mRNA transport into cytoplasm
20	kmt*Bax_mRNA	Bax_mRNA transport into cytoplasm
21	kmt*PIDD_mRNA	PIDD_mRNA transport into cytoplasm
22	kmt*p21_mRNA	p21_mRNA transport into cytoplasm
23	f0*ATM-P*Wip1	ATM-P + Wip1 → ATM-P:Wip1
24	r0*ATM-P:Wip1	ATM-P:Wip1 → ATM-P + Wip1
25	k0* ATM-P:Wip1	ATM-P:Wip1 → deg_ATM-P + Wip1
26	f1*ATM-P*p53	ATM-P + p53 → ATM-P:p53
27	r1*ATM-P:p53	ATM-P:p53 → ATM-P + p53
28	k1*ATM-P:p53	ATM-P:p53 → ATM-P + p53-P
29	f1*p53-P*PKCδ	p53-P + PKCδ → p53-P:PKCδ
30	r1*p53-P:PKCδ	p53-P:PKCδ → p53-P + PKCδ
31	k1*p53-P:PKCδ	p53-P:PKCδ → p53-PP + PKCδ
32	f2*p53*Mdm2	p53 + Mdm2 → p53:Mdm2
33	r2*p53:Mdm2	p53:Mdm2 → p53 +Mdm2
34	k2*p53:Mdm2	p53:Mdm2 → deg_p53 + Mdm2
35	f2p*p53-P*Mdm2	p53-P + Mdm2 → p53-P:Mdm2
36	r2p*p53-P:Mdm2	p53-P:Mdm2 → p53-P + Mdm2
37	k2p*p53-P:Mdm2	p53-P:Mdm2 → deg_p53-P + Mdm2
38	f3*ATM-P*Mdm2	ATM-P + Mdm2 → ATM-P:Mdm2
39	r3*ATM-P:Mdm2	ATM-P:Mdm2 → ATM-P + Mdm2

40	k3*ATM-P:Mdm2	ATM-P:Mdm2 → ATM-P + deg_Mdm2
41	(sDSB:MRN+cDSB:MRN)*f4*ATM*ATM-P	ATM + ATM-P → ATM:ATM-P
42	(sDSB:MRN+cDSB:MRN)*r4*ATM:ATM-P	ATM:ATM-P → ATM + ATM-P
43	k4*ATM:ATM-P	ATM:ATM-P → 2ATM-P
44	f6*p53-PP*Mdm2	p53-PP + Mdm2 → p53-PP:Mdm2
45	r6*p53-PP:Mdm2	p53-PP:Mdm2 → p53-PP + Mdm2
46	k6*p53-PP:Mdm2	p53-PP:Mdm2 → deg_p53-PP + Mdm2
47	s_bind*MRN*sDSB	sDSB + MRN → sDSB:MRN
48	s_dis*sDSB:MRN	sDSB:MRN → sDSB + MRN
49	s_rep*sDSB:MRN	sDSB:MRN → fixed_DSB + MRN
50	c_bind*MRN*cDSB	cDSB + MRN → cDSB:MRN
51	c_dis*cDSB:MRN	cDSB:MRN → cDSB + MRN
52	c_rep*cDSB:MRN	cDSB:MRN → fixed_DSB + MRN
