# Supplementary material for: Stochasticity of Intranuclear Biochemical Reaction Processes Controls the Final Decision of Cell Fate Associated with DNA Damage
Source: PLoS One. 2014 Jul 8;9(7):e101333. doi: 10.1371/journal.pone.0101333 (PMC4086823; doi:10.1371/journal.pone.0101333)
Supplement: Table S4 — Initial conditions of cytoplasmic species. (PDF) [file pone.0101333.s010.pdf]

Table S4 Initial conditions of cytoplasmic species

Species	Value (nM)	Description
cBax_mRNA	1	Bax mRNA in cytoplasm
Bax_c	0.6	Bax protein in cytoplasm
Bax_m	0	Bax protein in mitochondria
cBcl-2_mRNA	1.5	Bcl-2 mRNA in cytoplasm
Bcl-2_c	26.7	Bcl-2 protein in cytoplasm
Bcl-2:Bax	0	Complex of Bcl-2_c with Bax_c
Bcl-2:Bax_m	0	Complex of Bcl-2_c with Bax_m
cp21_mRNA	0	p21 mRNA in cytoplasm
p21_c	0	p21 protein in cytoplasm
PIDD_mRNA	0	p53-induced protein with a death domain (PIDD) mRNA in cytoplasm
PIDD_c	0	PIDD protein in cytoplasm
Proc-2	30	Procaspase (Proc)-2 which is the inactive form
PIDD:Proc-2	0	Complex of PIDD with Proc-2
Casp-2	0	Caspase (Casp)-2 which is the active form
Bid	25	BH3 interacting domain death agonist (Bid)
Casp-2:Bid	0	Complex of Casp-2 with Bid
tBid	0	Truncated Bid (tBid)
Bax:tBid	0	Complex of Bax_c with tBid
Bcl-2:Bax:tBid	0	Complex of Bcl-2_c and Bax:tBid
M_c	10	The state that there are no pores in mitochondrial outer membrane
M_o	0	The state that there are pores in mitochondrial outer membrane
Cytc_m	10000	Cytochrome *c* (Cytc) in mitochondria
Cytc_r	0	Cytc released from mitochondria, but remaining in M_o
Cytc_c	0	Cytc in cytoplasm
M_o:Cytc_m	0	The state that Cytc binds to pores in mitochondrial outer membrane
SMAC_m	100	Second mitochondria-derived activator of caspase (SMAC) in mitochondria
SMAC_r	0	SMAC released from mitochondria, but remaining in M_o
SMAC_c	0	SMAC in cytoplasm
M_o:SMAC_m	0	The state that SMAC binds to pores in mitochondrial outer membrane
Apaf1	370	Apoptotic protease-activating factor-1 (Apaf-1)
ATP	10	Adenosine triphosphate (ATP)
Apop	0	Apoptosome complex containing Cytc, ATP and Apaf-1
Proc-9	30	Proc-9 which is the inactive form
Apop:Proc-9	0	Complex of Apoptosome with a Proc-9
Apop:Proc-9_2	0	Complex of Apoptosome with two Proc-9

Casp-9	0	Casp-9 which is the active form
Proc-3	200	Proc-3 which is the inactive form
Casp-9:Proc-3	0	Complex of Casp-9 with Proc-3
Casp-3	0	Casp-3 which is the active form
p21:Casp-3	0	Complex of p21_c with Casp-3
p21:Proc-3	0	Complex of p21_c with Proc-3
XIAP	60	X-linked inhibitor of apoptosis protein in cytoplasm (XIAP)
Casp-3:XIAP	0	Complex of Casp-3 with XIAP
Proc-9:XIAP	0	Complex of Proc-9 with XIAP
SMAC_XIAP	0	Complex of SMAC_c with XIAP
