# Supplementary material for: Stochasticity of Intranuclear Biochemical Reaction Processes Controls the Final Decision of Cell Fate Associated with DNA Damage
Source: PLoS One. 2014 Jul 8;9(7):e101333. doi: 10.1371/journal.pone.0101333 (PMC4086823; doi:10.1371/journal.pone.0101333)
Supplement: Table S5 — Ordinary differential equations used for each cytoplasmic reaction. (PDF) [file pone.0101333.s011.pdf]

Table S5 Ordinary differential equations used for each cytoplasmic reaction

d cBax_mRNA /dt=-d_mbax*cBax_mRNA

d Bax_c /dt=s_bax*cBax_mRNA -d_bax*Bax_c -k1f*Bax_c*tBid +k1r*Bax:tBid -k13f*Bcl-2_c*Bax_c +k13r*Bcl-2:Bax

d Bax_m /dt=-d_bax*Bax_m +k1c*Bax:tBid -k13f*Bcl-2_c*Bax_m +k13r*Bcl-2:Bax_m

d cBcl-2_mRNA /dt=-d_mbcl*cBcl-2_mRNA

d Bcl-2_c /dt=s_bcl*cBcl-2_mRNA -d_bcl*Bcl-2_c -k13f*Bcl-2_c*Bax_c +k13r*Bcl-2:Bax -k13f*Bcl-2_c*Bax:tBid +k13r*Bcl-2:Bax:tBid -k13f*Bcl-2_c*Bax_m +k13r*Bcl-2:Bax_m

d Bcl-2:Bax /dt=-d_bcl*Bcl-2:Bax +k13f*Bcl-2_c*Bax_c-k13r*Bcl-2:Bax

d Bcl-2:Bax_m /dt=-d_bcl*Bcl-2:Bax_m +k13f*Bcl-2_c*Bax_m-k13r*Bcl-2:Bax_m

d cp21_mRNA /dt=-d_mp21*cp21_mRNA

d p21_c /dt=s_p21*cp21_mRNA -d_p21*p21 -k12f*p21*Proc-3 +k12r*p21:Proc-3 -k18f*p21*Casp-3 +k18r*p21:Casp-3

d PIDD_mRNA /dt=-d_mpid*cPIDD_mRNA

d PIDD_c /dt=s_pidd*cPIDD_mRNA -d_pidd*PIDD_c -k14f*PIDD_c*Proc-2+k14r*PIDD:Proc-2 +k15*PIDD:Proc-2

d Proc-2 /dt=s_pro2 -d_pro2*Proc-2 -k14f*PIDD_c*Proc-2+k14r*PIDD:Proc-2

d PIDD:Proc-2 /dt=k14f*PIDD_c*Proc-2 -k14r*PIDD:Proc-2 -k15*PIDD:Proc-2

d Casp-2 /dt=-d_pro2*Casp-2 +k15*PIDD:Proc-2 -k16f*Casp-2*Bid+k16r*Casp-2:Bid +k17*Casp-2:Bid

d Bid /dt=s_bid -d_bid*Bid -k16f*Casp-2*Bid+k16r*Casp-2:Bid

d Casp-2:Bid /dt=k16f*Casp-2*Bid-k16r*Casp-2:Bid -k17*Casp-2:Bid

d tBid /dt=-d_bid*tBid +k17*Casp-2:Bid -k1f*Bax_c*tBid+k1r*Bax:tBid+k1c*Bax:tBid

d Bax:tBid /dt=k1f*Bax_c*tBid-k1r*Bax:tBid-k1c*Bax:tBid -k13f*Bcl-2_c*Bax:tBid+k13r*Bcl-2:Bax:tBid

d Bcl-2:Bax:tBid /dt=k13f*Bcl-2_c*Bax:tBid-k13r*Bcl-2:Bax:tBid

d M_c /dt=-(mf3*Bax_m^4*M_c)/(mJ3^4+Bax_m^4) +mr3*M_o

d M_o /dt=(mf3*Bax_m^4*M_c)/(mJ3^4+Bax_m^4) -mr3*M_o -mf1*M_o*SMAC_m +mr1*M_o:SMAC_m +mk1*M_o:SMAC_m -mf1*M_o*Cytc_m +mr1*M_o:Cytc_m+mk1*M_o:Cytc_m

d Cytc_m /dt=-mf1*M_o*Cytc_m+mr1*M_o:Cytc_m

d Cytc_r /dt=mk1*M_o:Cytc_m -mk2*Cytc_r+mk2*Cytc_c

d Cytc_c /dt=mk2*Cytc_r-mk2*Cytc_c -k5f*Cytc_c*Apaf-ATP+k5r*Apop

d M_o:Cytc_m /dt=mf1*M_o*Cytc_m-mr1*M_o:Cytc_m-mk1*M_o:Cytc_m

d SMAC_m /dt=-mf1*M_o*SMAC_m+mr1*M_o:SMAC_m

d SMAC_r /dt=mk1*M_o:SMAC_m -mk2*SMAC_r+mk2*SMAC_c

d SMAC_c /dt=mk2*SMAC_r-mk2*SMAC_c -k4f*SMAC_c*XIAP+k4r*XIAP:SMAC

d M_o:SMAC_m /dt=mf1*M_o*SMAC_m-mr1*M_o:SMAC_m-mk1*M_o:SMAC_m

d Apaf-1 /dt=-k5f*Cytc_c*Apaf-ATP+k5r*Apop

d ATP /dt=-k5f*Cytc_c*Apaf-ATP+k5r*Apop

d Apop /dt=k5f*Cytc_c*Apaf-ATP-k5r*Apop -k6f*Apop*Proc-9+k6r*Apop:Proc-9

d Proc-9 /dt=-k6f*Apop*Proc-9+k6r*Apop:Proc-9 -k7f*Apop:Proc-9*Proc-9+k7r*Apop:Proc-9_2 -k11f*Proc-9*XIAP +k11r*XIAP:Proc-9

d Apop:Proc-9 /dt=k6f*Apop*Proc-9-k6r*Apop:Proc-9 -k7f*Apop:Proc-9*Proc-9+k7r*Apop:Proc-9_2 +k8*Apop:Proc-9_2

d Apop:Proc-9_2 /dt=k7f*Apop:Proc-9*Proc-9 -k7r*Apop:Proc-9_2 -k8*Apop:Proc-9_2

d Casp-9 /dt=k8*Apop:Proc-9_2 -k9f*Casp-9*Proc-3+k9r*Casp-9:Proc-3 +k10*Casp-9:Proc-3

d Proc-3 /dt=-k9f*Casp-9*Proc-3+k9r*Casp-9:Proc-3 -k12f*p21*Proc-3+k12r*p21:Proc-3

d Casp-9:Proc-3 /dt=k9f*Casp-9*Proc-3-k9r*Casp-9:Proc-3 -k10*Casp-9:Proc-3

d Casp-3 /dt=k10*Casp-9:Proc-3 -k12f*Casp-3*XIAP+k12r*XIAP:Casp-3 -k18f*p21*Casp-3 +k18r*p21:Casp-3 +k19*p21:Casp-3

d p21:Casp-3 /dt=k18f*p21*Casp-3-k18r*p21:Casp-3-k19*p21:Casp-3

d p21:Proc-3 /dt=k12f*p21*Proc-3-k12r*p21:Proc-3

d XIAP /dt=-k4f*SMAC_c*XIAP+k4r*XIAP:SMAC -k11f*Proc-9*XIAP+k11r*XIAP:Proc-9 -k12f*Casp-3*XIAP +k12r*XIAP:Casp-3

d Casp-3:XIAP /dt=k12f*Casp-3*XIAP-k12r*XIAP:Casp-3

d Proc-9:XIAP /dt=k11f*Proc-9*XIAP-k11r*XIAP:Proc-9

d SMAC_XIAP /dt=k4f*SMAC_c*XIAP-k4r*XIAP:SMAC
