# Supplementary material for: Stochasticity of Intranuclear Biochemical Reaction Processes Controls the Final Decision of Cell Fate Associated with DNA Damage
Source: PLoS One. 2014 Jul 8;9(7):e101333. doi: 10.1371/journal.pone.0101333 (PMC4086823; doi:10.1371/journal.pone.0101333)
Supplement: Text S1 — Detailed numerical calculation method. (PDF) [file pone.0101333.s012.pdf]

Text S1 Detailed numerical calculation method

Effect of the proportion of simple and complex DSB on the distributions

An IR dose of 1 Gy produced 20–40 DSBs per cell, and the distribution of DSB generation followed a Poisson distribution [1]. DSBs generated by IR irradiation were classified into simple DSB (sDSB) and complex DSB (cDSB), with the condition that the incidences of sDSB and cDSB were 60-80% and 20-40%, respectively [2]. We calculated the distribution of each DSB with various proportions to investigate the effect of the proportions on distribution. Initially, the number of total DSB was sampled from Poisson distribution with mean value of 35*IR-dose (Gy), then, total DSB was divided into sDSB and cDSB. The proportion of sDSB was fixed to 70%, uniform random variables between 60% and 80% (mean value is 70%), or normal random variables whose mean value is 70%. The calculated distributions of each DSB at IR-dose of 0.3, 2.5 and 6.0 Gy were shown in Figure S1-S3. The distributions of each DSBs in fixed, uniform and normal proportions were not much different (Figure S1-S3). Therefore, we selected fixed proportion in the proposed model in order to reduce calculation costs.

Direct Hybrid Method

Our proposed model describes the generation of IR-induced DSBs, the DSB repair system, and the p53 signaling network as the nuclear reactions (Figure 2), and the apoptosis induction pathway as the cytoplasmic reactions (Figure 3). We simulate the nuclear and cytoplasmic reactions as stochastic and deterministic processes (hybrid simulation), respectively. In the proposed model, nuclear species were described by the number of molecules (N), whereas the cytoplasmic species were described by concentration (nM). Therefore, the hybrid simulation required the translation of the species that translocate from the nucleus to the cytoplasm (p21_mRNA, Bax_mRNA, Bcl2_mRNA and PIDD_mRNA in our proposed model) with using the following equation:

$$C = \frac{N}{N_A V_c} \qquad \text{Eq.1}$$

where, C and N represented concentration (nM) and number of molecules (N) of chemical species in interest, respectively. Moreover, N_A and V_c represented the Avogadro constant and the cytoplasmic volume (pL), respectively. Ciliberto *et al.* estimate that the volume of a cell is 2.09 pL and that the ratio of volume of cytoplasm to that of nucleus is equal to 15 in their model [3]. Accordingly, we assumed that cytoplasmic volume (V_c) is 1.96 pL. The hybrid simulation of the proposed model was executed employing the “Direct Hybrid Method” proposed by Alfonsi *et al.* [4], and the procedures were as follows.

Step1. Set t=0. Generation of DSBs. Total DSB was randomly sampled from a Poisson distribution that had a mean of 35×IR-dose (Gy). The total DSB was then partitioned and set as follows: simple DSB=0.7*Total DSB and complex DSB=0.3*Total DSB. Set the initial conditions of other species (Table S2 and S4).

Step2. Generate the random variable ξ =Exp(1); Denote by Exp(1) the exponential random variable of parameter 1.

Step3. Increase time from $t=t$ to $t=t+\Delta t$.

Step4. Solve the system of ordinary differential equations described in Table S5 (cytoplasmic reactions). Convert concentrations to number of molecules (p21_mRNA, Bax_mRNA, Bcl2_mRNA and PIDD_mRNA according to Eq.1).

Step5. Calculate propensities of each nuclear reaction (a_j) described in Table S3. Solve the cumulative propensities (G_σ) as follows.

$$\frac{dG_\sigma}{dt}(t) = \sum_{j=1}^{n} a_j(X(t),t)$$

where, n is the number of nuclear reactions and $X(t)$ represents the chemical species included in the proposed model.

Step6. If G_σ was less than a random variable ξ, go to step 9. If not, go to step 7.

Step7. Determine the reaction to be fired by "Gillespie direct method" [5]. Update the number of all molecules in nucleus according to the fired reaction.

Step8. Set $G_\sigma = 0$. Generate the random variable ξ =Exp(1).

Step9. Convert the number of molecules to concentrations (p21_mRNA, Bax_mRNA, Bcl2_mRNA and PIDD_mRNA according to Eq. 1). Loop to Step 3.

Model parameters

Initial conditions and kinetic parameters in the proposed model were shown in Tables S1, S2, S4. In mammalian cells, typical translation rate was four amino acids per second [6]. Based on this biological findings, we assumed synthetic rates of several species in the proposed model (Table S1). Half-lives of Bcl-2 and p21 mRNA were measured by Kren et al. [7]. We calculated degradation rates of Bcl-2 and p21 mRNA from the experimental data, and degradation rates of other mRNA were estimated in this research (Table S1). Other parameters were set to our estimated or conventional model's values [8-10]. Initial conditions in intrinsic apoptosis pathway model (part of ODE in the proposed model) were based on the conventional models [9,10] (Table S4). Initial conditions of other species were our estimated values (Table S2).

References

1. Rothkamm K, Lobrich M (2003) Evidence for a lack of DNA double-strand break repair in human cells exposed to very low x-ray doses. *Proc Natl Acad Sci USA* 100: 5057–5062.

2. Stewart RD (2001) Two-lesion kinetic model of double-strand break rejoining and cell killing. *Radiat Res* 156: 365–378.

3. Ciliberto A, Novak B, Tyson JJ (2005) Steady states and oscillations in the p53/Mdm2 network. *Cell Cycle* 4: 488–493.

4. Alfonsi A, Cances E, Turinici G, Di Ventura B, Huisinga W (2005) Adaptive simulation of hybrid stochastic and deterministic models for biochemical systems. *ESAIM PROC* 14: 1–13.

5. Gillespie DT (1976) A general method for numerically simulating the stochastic time evolution of coupled chemical reactions *J Comp Phys* 22: 403-434.

6. Audibert, A, Weil, D, Dautry, F. (2002) In Vivo Kinetics of mRNA Splicing and Transport in Mammalian Cells. *Mol Cell Biol* 22(19): 6706-6718.

7. Kren, BT, Steer, CJ, (1996) Posttranscriptional regulation of gene expression in liver regeneration: role of mRNA stability. *EMBO J* 10:819-828.

8. Ma L, et al. (2005) A plausible model for the digital response of p53 to DNA damage. *Proc Natl Acad Sci USA* 102: 14266–14271.

9. Hua F, Cornejo MG, Cardone MH, Stokes CL, Lauffenburger DA (2005) Effects of Bcl-2 levels on Fas signaling-induced caspase-3 activation: molecular genetic tests of computational model predictions. *J Immunol* 75(2): 985-995.

10. Albeck JG, Burke JM, Spencer SL, Lauffenburger, DA, Sorger PK (2008) Modeling a snap-action, variable-delay switch controlling extrinsic cell death. *PLoS Biol* 6: e299.
